# Potentially Harmful Element Concentrations in the Vegetables Cultivated on Arable Soils, with Human Health-Risk Implications

**DOI:** 10.3390/ijerph16204053

**Published:** 2019-10-22

**Authors:** Agnieszka Gruszecka-Kosowska

**Affiliations:** AGH University of Science and Technology, Faculty of Geology, Geophysics and Environmental Protection, Department of Environmental Protection, Al. Mickiewicza 30, Kraków 30-059, Poland; agnieszka.gruszecka@agh.edu.pl

**Keywords:** human health risk assessment, edible plant, agricultural soil, potentially harmful element, transfer factor, dietary intake

## Abstract

Potentially harmful elements (PHEs) were investigated in eight groups of vegetables cultivated in southern Poland and the relevant health-risk implications were assessed. The PHE contents belonged to the following ranges (mg/kg wet weight) in edible parts: As < limit of detection (LOD)-0.056, Cd < LOD–0.375, Co < LOD–0.029, Cu < LOD–7.638, Hg < LOD–0.163, Ni < LOD–0.299, Pb < LOD–0.580, Sb < LOD–0.163, Tl < LOD–0.128, and Zn 1.23–34.9. The PHE concentrations decreased in the following order: Zn > Cu > Ni > Cd > Pb > Sb > Hg > Tl > As > Co. The concentrations of essential PHEs decreased as follows: root > leaf > seed > tuber > legume > inflorescence > shoot > fruit, while the unnecessary PHEs followed this sequence: leaf > root > tuber > legume > inflorescence > seed > shoot > fruit. Soil-to-plant transfer factors revealed capacities to adsorb Cd, Hg, and Tl in roots; Cd, Hg, Tl, and Zn in leaves; Cd, Hg, and Sb in tubers; and Cu, Sb, and Zn in legumes and seeds. The daily intake rates, as a percentage of permissible maximum tolerable daily intake, amounted to the following proportions: Cd 23%, Tl 13%, Hg 5.0%, Ni 3.1%, Pb 2.6%, and As 0.4%. Non-carcinogenic risk described as hazard quotient (HQ) was exceeded in root (HQ = 12.1), leafy (HQ = 2.1), and tuber (HQ = 1.4) vegetables. The carcinogenic risk of As (CR = 8.54 × 10^−5^) was found unacceptable. The margins of exposure for adults (MOE = 3.1) and children (MOE = 1.6), respectively, indicated a low health risk of Pb in consumed vegetables.

## 1. Introduction

The trend of fresh vegetable consumption became more and more popular recently. For adults, the recommended daily intake of vegetables is 300 g per person [1]. However, the World Health Organization (WHO) recommends eating >400 g of fruit and vegetables per day to improve overall health and reduce the risk of certain non-communicable diseases (NCDs) [2]. On the other hand, contamination of the food chain, leading to potential health risks, is one of the major environmental pathways of human exposure to potentially harmful elements (PHEs) [3]. Thus, more and more studies have been conducted on the contamination of edible plants with PHEs and the related health-risk implications [3,4,5,6,7,8,9,10].

PHEs adverse health effects are reported for many years. Some elements are essential to human body (i.e., Cu and Zn), but when they are consumed in too high quantities they cause toxic effects. On the other hand, some PHEs (i.e., As, Cd, and Pb) are considered toxic to humans regardless the dose taken [11]. Furthermore, as reported in the literature, vegetables accumulate higher levels of PHEs comparing to other edible plants [12,13].

Agriculture is common in Poland, as arable lands constitute about 73% of farmlands [14]. Moreover, intense mining and processing of coal [15,16], as well as of Zn–Pb [17,18] and Cu ores [19], caused serious environmental pollution with PHEs, especially in southern Poland. Previous studies performed in the Region of Upper Silesia in the 1990s indicated a high contamination of soil with Cd, Pb, and Zn by industrial emissions [20]. Besides, the excess of such PHE contents in soils and root vegetables was established by comparing the related amounts to the quantities found in other agricultural regions and the maximum allowable concentrations [21], respectively. Recent investigations, however, revealed a lack of excess of the permissible concentration levels of heavy metals and metalloids in arable soils of southern Poland [22]. Nevertheless, it has been concurrently indicated that some of the investigated PHEs (i.e., Cd and Zn) were mobile and bioavailable [23]. 

Moreover, PHE contents in soil may origin not only form the bedrock itself. More and more significant are anthropogenic sources i.e., illegal waste deposits, agricultural inputs as pesticides and inorganic fertilizers, industrial effluents, and fallout of industrial and urban emissions [13,24]. All of them may have consequences for food quality.

Thus, since vegetables should be consumed in significant amounts and fresh edible plants cultivated on site are sold locally, even low concentrations of PHEs in food may cause health risk, also due to long-term consumption. Taking the above into consideration, the determination of selected PHE concentrations in different groups of vegetables, cultivated in recently investigated arable soils of southern Poland [22,23], with the health-risk implication assessment, was adopted as the goal of the present study. Detailed objectives of the study included the determination of the following: (1) As, Cd, Co, Cr, Cu, Hg, Ni, Pb, Sb, Se, Tl, and Zn contents in 36 types of vegetables, (2) soil-to-plant transfer indices, (3) contribution of PHEs to the daily intake rates via consumption of vegetables, and (4) human health-risk assessment from the PHE contents in consumed vegetables.

## 2. Materials and Methods

### 2.1. Vegetable Sampling and Preparation

**Figure 1:**
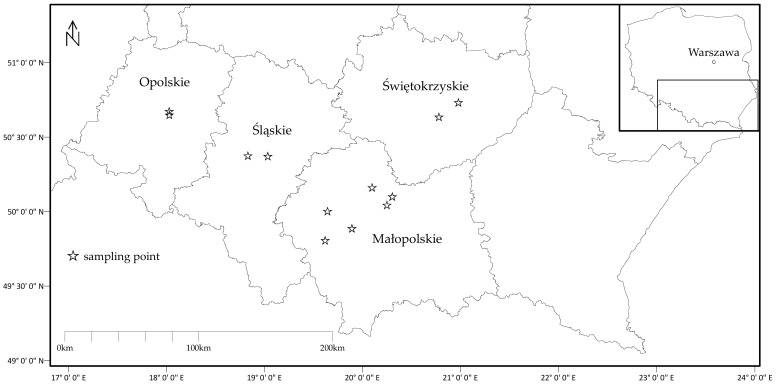
Vegetable sampling site locations in the Regions of southern Poland (modified from [22]).

Vegetable samples were collected from the produce bought on the local “fresh food markets” on the same sites where previously arable soil samples had been investigated, in the following Regions of southern Poland: Opolskie, Śląskie, Małopolskie, and Świętokrzyskie (Figure 1). During the growing seasons of 2015 and 2016, 36 types of vegetables (12 leafy, 7 root, 5 fruits, 4 tuber, 4 seed, 2 inflorescence, 1 shoot, and 1 legume) were collected (Table 1). Altogether, 206 vegetable samples were analyzed. After transportation to the laboratory, vegetable samples were prepared as is for consumption: They were washed and peeled. The vegetables were cut into small pieces, placed on open porcelain dishes and dried under radiant lamps at 70 °C. Dried vegetable samples were next ground into a coarse powder with a coffee grinder and stored in sealed bags for further analysis. The grinder was cleaned after each course of vegetable sample processing to prevent cross-contamination.

### 2.2. Sample Analyses

Vegetable samples (0.5 g, accurate to 0.001 g) were weighed and placed in mineralization flasks, with 15 cm^3^ of HNO_3_ and 5 cm^3^ of H_2_O_2_ added. The samples were left for the organic matter to decompose overnight. The next step consisted in digestion in the SCP Science DigiPREP HT High Temperature Digestion System (SCP Science, Quebec, Canada) at 130 °C for 2 h. After cooling down the extract solutions, their volume was expanded to 50 cm^3^ by adding ultrapure water. At the same time, blank and reference samples (white cabbage, Certified Reference Material BCR^®^-679) were prepared. The total concentrations of PHEs (As, Cd, Co, Cr, Cu, Hg, Ni, Pb, Sb, Se, Tl, and Zn) were determined by inductively coupled plasma-mass spectrometry (ICP-MS) (ELAN 6100; Perkin Elmer, Waltham, MA, USA), according to United States Environmental Protection Agency USEPA 6020B protocol [25] and International Agency for Standardization ISO protocol [26].

All the results of the PHE concentrations in vegetables were determined as dry mass [13,27,28]. The water content in analyzed vegetables was not investigated. However, for the purpose of estimating daily intakes in risk assessment analyses and to facilitate comparison to guidance values on European Union (EU) food standards PHE contents in vegetables were referred to wet weight (ww.), as recommended by the USEPA in accordance with Equation (1). Water contents in particular plant samples were taken from the USEPA exposure factors book [29]:

c_ww_ = c_dw_ × (100 − w)/100 (1)

where: c_ww_—concentration of PHE in plant sample wet weight; c_dw_—concentration of PHE in plant sample dry weight; w—percentage water content.

### 2.3. Quality Control

Vegetable samples analyses were performed, maintaining the standard certified analytical quality control procedure [30]. To achieve impartial and unequivocal ICP-MS results, the elements were also measured, using inductively coupled plasma-optical emission spectroscopy (ICP-OES) (OPTIMA 7300DV; Perkin Elmer, Waltham, MA, USA), according to USEPA 6020B protocol [25] and ISO protocol [31]. The certified reference material CRM (white cabbage, Certified Reference Material BCR^®^-679) was analyzed at the same time. Recovery from CRM plant was established between 83% and 124% for the majority of analyzed PHEs. In this method, reagent blanks and duplicates were used to ensure quality assurance and quality control. All the reagents used in the laboratory analysis were analytically pure. The results of sample investigations were contained within the allowable error change values. Analytical bias was statistically insignificant (*p* = 0.05). The accuracy of ICP-MS and ICP-OES systems was satisfactory and verified by six different solution injections. Rh was used as an internal standard. In the ICP-MS analysis, element correction equations were applied to each element to minimize the impact of interferences. The limit of detection (LOD) values of the investigated PHEs were as follows (µg/L): As < 0.001, Cd < 0.0005, Co < 0.0005, Cr < 0.0005, Cu < 0.0005, Hg < 0.001, Ni < 0.002, Pb < 0.0005, Sb < 0.0005, Se < 0.002, Tl < 0.001, and Zn < 0.001.

### 2.4. Statistical Analyses

Statistical analysis involved determination of mean, standard deviation, and minimum and maximum values using a Microsoft Excel 2007 spreadsheet. The software package STATISTICA 13 (TIBCO Software Inc., Palo Alto, CA, USA) was used for further statistical analysis. To check data distribution the Shapiro–Wilk’s test (*p* = 0.05) was used. The one-way ANOVA at the 95% confidence level was used to check for any significant differences (*p* ≤ 0.05) between the average PHE concentrations and bioconcentration factors among analyzed groups of vegetables and different sampling regions. On such basis the scientific hypotheses were tested as follows: H_0_: content of individual PHEs and bioconcentration factors values were not conditioned by the part of vegetable/sampling region; H_1_: content of individual PHEs and bioconcentration factors values were conditioned by the part of vegetable/sampling region. If differences were found to be significant post-hoc analysis were performed followed by the Fisher’s Least Significant Difference (LSD) test. Hierarchical cluster analysis (HCA) and principal components analysis (PCA) were performed for multivariate statistical modeling of the input data. Halves of the limit of detection (LOD) values were assigned to undetected results in all statistical analysis that were carried out, as recommended by the WHO [32].

### 2.5. Soil-To-Plant Transfer Indices

The transfer factor is commonly used in environmental geochemistry to determine translocation of PHEs in soil and plant system [33]. Two soil-to-plant transfer factors were calculated in the current research in order to determine environmental bioavailability of PHEs. The bioaccumulation coefficient (BA) described the transference of PHE from the soil to the plant, while the bioconcentration coefficient (BC) described the plant capacity to adsorb PHE from the soil when PHE occurred in an available form [34]. The BA and BC values were calculated for the investigated groups of vegetables, respectively, from general Equations (2) and (3) [35]:

BA = C_veg_/C_st_ (2)

BC = C_veg_/C_sa_ (3)

where C_veg_ is the mean concentration of particular PHE (mg/kg ww.) in the group of investigated vegetables; C_st_ is the mean total concentration of particular PHE determined in soil samples (mg/kg dw.) from southern Poland, using *aqua regia* digestion [22]; and C_sa_ is the mean available concentration of particular PHE determined in soil samples (mg/kg dw.) from southern Poland after extraction with (i) 0.11 mol/L CH_3_COOH (the first fraction of the BCR sequential extraction procedure F1) and (ii) 0.05 mol/L Na_2_EDTA [23].

### 2.6. Human Health Risk Assessment

The point estimate method developed by USEPA [36] was applied to assess the Human Health Risk Assessment (HHRA) arising from the consumption of PHEs in vegetables cultivated in southern Poland. The following parameters for both adults and children, using mean and 95th percentile (P95) of the PHE concentrations in vegetables, were calculated: The daily intake rate (DIR) values for particular PHEs were calculated as the sum of consumed vegetable groups, according to Equation (4) [37]:

DIR = Σ (C_veg_ × IR_veg_/BW) (4)

where C_veg_ is the concentration of particular PHE in the group of vegetables (mg/kg ww.); IR_veg_ is the vegetable ingestion rate (g ww./person-day) in the group of vegetables; BW is the body weight: 70 kg for adults and 15 kg for children [29].

The intake rates of the groups of vegetables assumed in this study were presented in Table 2. Three exposure scenarios of vegetable consumption were analyzed. The first scenario was designed for adults, based on the available Polish statistical data [38,39]. The second scenario was based on the vegetable intake rates recommended by USEPA [29], since the data on the consumption of certain vegetable groups were missing in the Polish statistical data. The third scenario was based on the intake values recommended by USEPA for children, since the Polish statistical data did not involve a subpopulation of children. 

The average daily doses (ADD) for PHE ingestion via consumed vegetables (mg/kg bw-d) were calculated as the sum of the consumed vegetable groups, using Equation (5):

ADD = Σ (C_veg_ × IR_veg_ × EF × ED × 10^−3^)/AT × BW (5)

where C_veg_ is the PHE concentration in the investigated group of vegetables (mg/kg ww.); IR_veg_ is the intake rate of vegetables (g ww./person-day); EF is the exposure frequency: 365 days/year; ED is the exposure duration: 30 years for adults and 6 years for children [40]; AT is the averaging time in days: ED × 365 for non-carcinogens and 70 years × 365 for carcinogens [40]; BW is body weight (kg), as in Equation (4); and 10^−3^ is the unit conversion factor. 

The non-carcinogenic PHE risk values from dietary exposure were calculated with Equation (6):

HQ = ADD/RfD(6)

where HQ is the hazard quotient and RfD is the reference dose for particular PHE. 

The RfD values were set to be as follows (mg/kg bw-day): As 3.00 × 10^−4^, Cd 1.00 × 10^−3^, Co 3.00 × 10^−4^, Cu 4.00 × 10^−2^, Hg 3.00 × 10^−4^, Ni 2.00 × 10^−2^, Sb 4.00 × 10^−4^, Tl 1.00 × 10^−5^, and Zn 3.00× 10^−1^ [41]. 

The total non-carcinogenic risk (HQ_t_) value for the investigated PHEs was calculated, using Equation (7):

HQ_t_ = HQ_1_ + HQ_2_ + … + HQ_n_ (7)

where HQ_s_ are the hazard quotient values for 1-n PHEs investigated in the study. 

The carcinogenic risk values of PHEs from dietary exposure were calculated, using Equation (8):

CR = ADD × SF_o_ (8)

where CR is the carcinogenic risk and SF_o_ is the oral slope factor for a particular PHE. 

Only As was considered as carcinogenic PHE in this study. There were no SF values available for other investigated elements at the time. The SF_o_ for As was set to be equal to 1.5 (mg/kg bw-day)^−1^ [41]. The total carcinogenic risk value, as the sum of partial CR values, was not calculated since As was the only carcinogenic PHE considered in this study.

The Pb risk of dietary exposure was calculated according to the margin of exposure (MOE) approach, as recommended by EFSA [42], using Equation (9) [43]:

MOE = BMDL/DIR(9)

where MOE is the margin of exposure value; BMDL is the benchmark dose (lower confidence limit), estimated at 1.2 µg/kg bw-day for adults and 0.6 µg/kg bw-day [44]; and DIR is the total amount of vegetables consumed daily under the analyzed intake scenarios.

## 3. Results and Discussion

### 3.1. Abundance of PHEs in Vegetables

In the 36 types of vegetables concentrations of twelve PHEs were investigated. Determined contents of Se and Cr concentrations were <LOD in all the analyzed samples. Consequently, those two PHEs were excluded from further analysis. Among the analyzed vegetables, the detectable rates (%) of the remaining investigated PHEs were as follows: As 21.8%, Cd 85.6%, Co 35.8%, Cu 98.5%, Hg 31.8%, Ni 7.35%, Pb 46.6%, Sb 41.7%, Tl 39.4%, and Zn 100%. 

A detailed record of statistical characteristics of the investigated PHE concentrations in the analyzed groups of vegetables is presented in Table 3. The concentrations of PHEs determined in the edible parts of vegetables were within the following ranges (mg/kg ww.): As < LOD–0.056, Cd < LOD–0.375, Co < LOD–0.029, Cu < LOD–7.638, Hg < LOD–0.163, Ni < LOD–0.299, Pb < LOD–0.580, Sb < LOD–0.163, Tl < LOD–0.128, and Zn 1.23–34.9. The results were consistent with the recent studies regarding Cd and Pb contents in the broccoli, cabbage, carrot, potato, parsley, celery, and beetroot investigated in the Region of Upper Silesia [27,45]. Contents of Cd, Pb, Cu, and Zn in parsley leaves and lettuce investigated in the region of South-Western Poland [46] were higher than observed in this study. It could be explained by the fact that leafy vegetables in the cited research were taken from the garden allotments located in highly industrialized cities, where permissible levels of PHEs were exceed, while vegetables investigated in the study were cultivated on arable soils fully suitable for vegetable cultivation [22]. 

The PHE concentrations determined in vegetables decreased in the following order: Zn > Cu > Ni > Cd > Pb > Sb > Hg > Tl > As > Co. The mean concentrations decreased in the analyzed groups of vegetables in the following sequences: As: root > leaf > tuber; Cd: leaf > root > tuber > legume > inflorescence > fruit; Co: leaf > legume > root > tuber = seed > inflorescence; Cu: seed > legume > leaf > tuber > root > inflorescence > fruit > shoot; Hg: legume > root > leaf > tuber; Ni: root > tuber; Pb: leaf > legume > tuber > root > shoot > fruit > seed; Sb: tuber > legume > seed > leaf > inflorescence > fruit > root; Tl: root > leaf > inflorescence > tuber > legume > seed; and Zn: seed > leaf > root > shoot > tuber > legume > inflorescence > fruit. If a section of a plant was not mentioned, the concentration of a particular PHE was <LOD there. Taking under consideration the essentiality of elements (i.e., Zn and Cu) or their potential essentiality for the plant (i.e., Co and Ni), the PHE concentrations in parts of vegetables decreased as follows: root > leaf > seed > tuber > legume > inflorescence > shoot > fruit. In reference to the unnecessary elements (As, Cd, Hg, Pb, Sb, and Tl), the decreasing concentration in parts of vegetables was determined as follows: leaf > root > tuber > legume > inflorescence > seed > shoot > fruit. The results of decreasing PHE contents achieved in this study, in parts of vegetables in particular, were consistent with the recent results obtained by other researchers [28,47,48,49,50,51,52,53]. Besides, metal content in plants strongly depends on soil properties [53]. However, a comparison of data between different studies should be done with caution due to time differences and methods applied. Soil remains the major source for accumulation of PHEs in plants [54]. PHEs uptake increases as their bioavailable contents increase in the soil [55]. The plant capacity to uptake PHEs differs for different PHEs and the same element might be accumulated at different ratio in various plant species [54]. 

Besides, metal content in plants strongly depends on soil properties [33,56]. Most of plants exclude excess bioavailable PHEs at their roots, where they are unable to interfere with physiological processes [54,57]. Nevertheless, leafy vegetables are considered as potential hyperaccumulators of PHEs [54,58]. When roots, tuber, and leaves with excessive contents of PHEs are consumed, they migrate in the food chain posing risk, which especially for humans, become an important topic of scientific research worldwide [44,59,60,61,62,63,64,65,66,67,68,69,70,71,72]. 

In order to determine the analyzed vegetable safety, the PHE content values were compared with the permissible levels stated in the EU regulation on maximum levels for certain contaminants in foodstuff [73] (Table 3). The maximum allowable concentrations were specified for Cd, Hg, and Pb. It was observed that mean concentrations were on the border of the maximum allowable concentrations (MAC) for Hg (MAC = 0.02 mg/kg ww.) in roots, leaves, and legumes, as well as for Cd in roots (MAC = 0.10 mg/kg ww.). In the case of the investigated groups of vegetables, mean concentrations were not close to the limit values for Pb (MAC = 0.3 mg/kg ww. in leaves and MAC = 0.1 mg/kg ww. in other plant parts).

### 3.2. Statistical Analyses

Principal component analysis (PCA) performed on PHEs concentrations in all 36 types of vegetables cultivated in southern Poland revealed four principal components (PCs) with eigenvalues >1, which explained 85% of the total variability observed (Table 4). The first principal component (PC1), which accounted for 32.7% of the variance, had high positive loading values (>0.5) for As, Cd, Co, Hg, Pb, and Tl. This positive correlation between parameters indicated that high concentration of these PHEs in edible plants was the consequence of the relatively high concentrations of these elements in soils. The second component (PC2) explained 19.5% of the variance and had high positive loading values for Cu and Zn, which corresponded with essentiality of these elements for plants (Figure S1). The third component (PC3) explained 16.4% of the variance and had high positive loading values for Ni and Tl that correlated with the highest observed concentrations of these elements in roots of edible plants. The fourth component (PC4) explained 16.7% of the variance and had positive loading values for Cd, Pb, and Sb, that could be correlated with the highest concentrations of these elements observed in leafy parts of edible plants.

Although, the results of the one-way ANOVA tests revealed no significant differences between the average concentrations for investigated PHEs in groups of vegetables (Table S1), hierarchical cluster analysis results explored similarities between concentrations of investigated PHEs in 36 types of edible plants. Hierarchical dendogram distinguished two similarity groups for the variables (basing on the restrictive Sneath’s criterion equal to 1/3 D_max_) (Figure S1). The first cluster could be associated with vegetables indicating much higher Zn (group Ia) and Cu (group Ib) concentrations in edible plant parts than in vegetables belonging to the second cluster. Vegetables located in the group I are known to be enriched in Zn and Cu, as they are essential elements [74,75]. The second cluster could be associated with vegetables revealing elevated concentrations of non-essential elements for plants, cumulated in different part of vegetables as a consequence of various factors i.e., bioavailable PHE concentrations in soil, soil properties, content of compounds affecting uptake by plants, mechanism of element uptake in plant itself [33,57,76].

Standardized contents of PHEs in all types of vegetables collected in four regions of southern Poland were presented for better visualization on the color scale map (Figure S2). The map revealed that the highest abundance of most of investigated PHEs comparing to other regions was observed in Śląskie region (except for Sb and Co). In the Małopolskie region the highest abundance values in edible plants were observed for Co, Sb, Pb, and Hg. In Opolskie region investigated plants were abundant in Sb, Hg, and Zn. In the Świętokrzyskie region the highest abundance was observed for As. The one-way ANOVA analysis revealed the significant differences in PHEs contents in vegetables from investigated regions as follows (Table S2). Cadmium contents in vegetables were significantly different in Śląskie than in Świętokrzyskie, Małopolskie, and Opolskie regions. Cobalt contents in vegetables were significantly different in Małopolskie than in Śląskie and Świętokrzyskie regions. Zinc contents in vegetables were significantly different in Opolskie than in other investigated regions. This regionalization might be explained by the geology of investigated regions. Śląskie and Małopolskie are the most industrialized from analyzed regions with long-term exploitation and processing of coal and Zn–Pb ores and additionally with the lowest soil pH and organic matter content from all investigated areas [22]. Location of Opolskie between regions: Śląskie on the east and Dolnośląskie on the west, which in turn is known for long-term exploitation and processing of Cu ores, might explain abundance of Opolskie region in Hg and Sb. In Świętokrzyskie region the lowest abundance in investigated PHEs was observed, as the area is known mainly for existence of sandstones and carbonate deposits. Moreover, geochemical background of PHEs in southern Poland is higher than elsewhere, especially of Zn, Pb, Cu, as well as accompanying elements i.e., As, Cd, Hg, Tl, and Ni, as a consequence of non-ferrous metals ores presence in these regions.

### 3.3. Soil-To-Plant Transfer Indices

**Figure 2:**
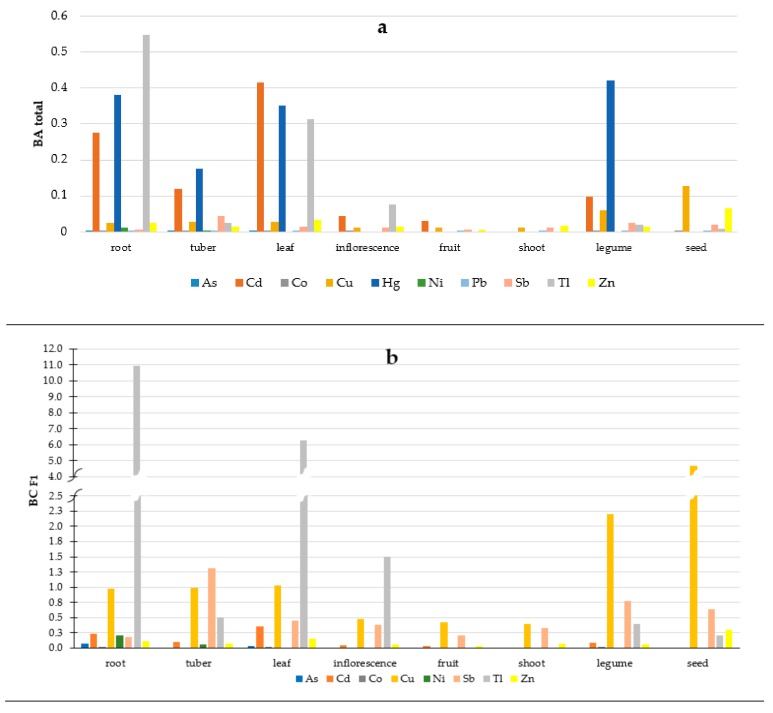

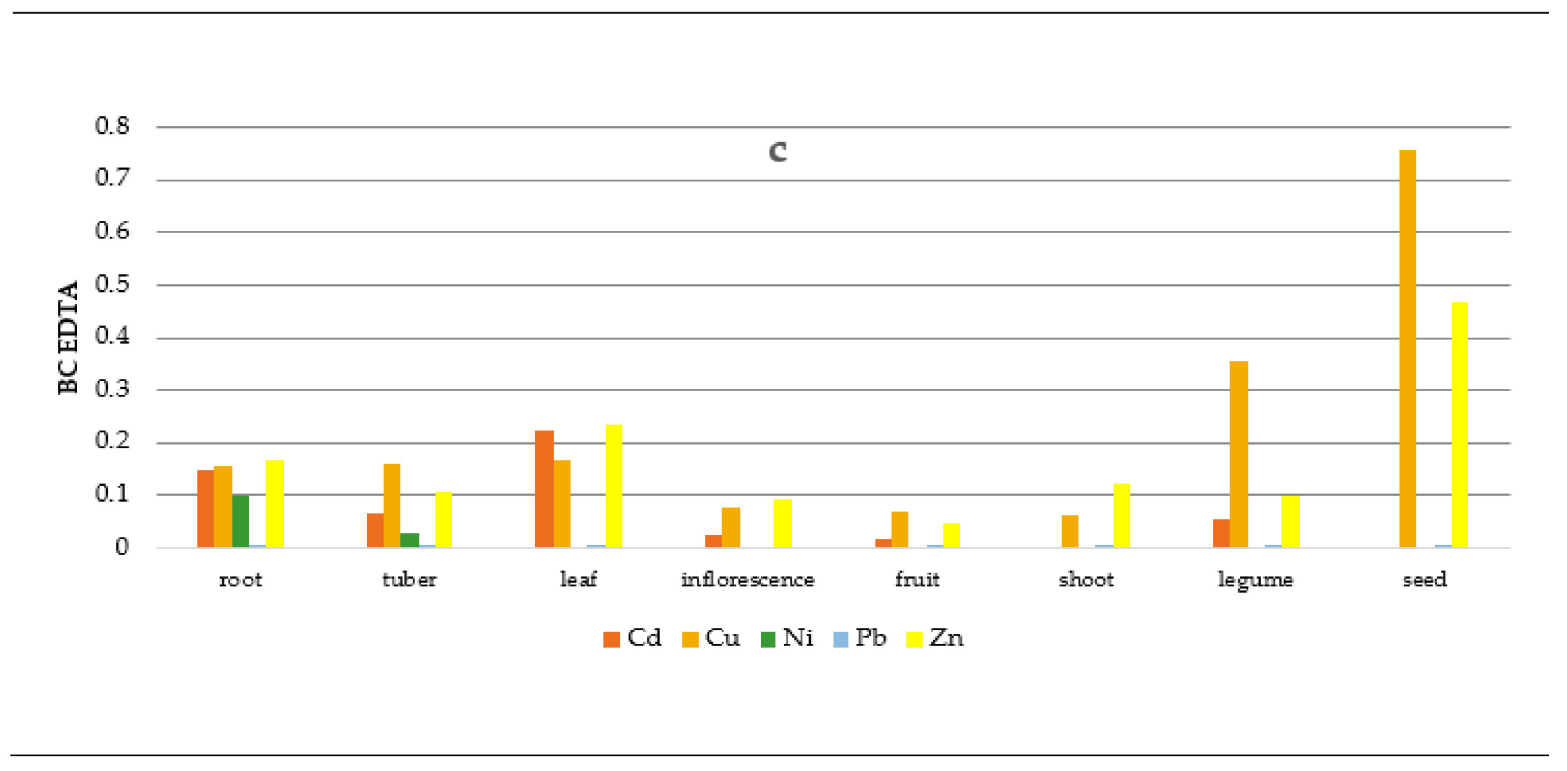
Soil-to-plant transfer indices for the analyzed groups of vegetables: (**a**) BA_total_—bioaccumulation coefficient, total PHE content, (**b**) BC_F1_—bioconcentration coefficient, PHE content of the F1 fraction of the BCR sequential extraction, (**c**) BC_EDTA_—bioconcentration coefficient, PHE content of 0.05 mol/L of the Na_2_EDTA extraction.

Soil is the main source of PHEs for plants. Elements taken up from soil are distributed to different parts of plants, and some of them are especially susceptible for PHEs accumulation. To define the efficiency of PHE uptake by vegetables the soil-to-plant transfer indices were calculated. The values of bioaccumulation coefficient (BA) and bioconcentration coefficient (BC) were presented in Figure 2. The bioconcentration coefficient (BC_F1_) values were not calculated for Hg and Pb, since the contents of those PHEs in soil samples had been <LOD after the first step of the BCR extraction. Besides, the bioconcentration coefficient (BC_EDTA_) values were not calculated for As, Co, Hg, Sb, and Tl, since those PHEs had not been determined in soil after the 0.05 mol/L Na_2_EDTA extraction. The bioaccumulation coefficient (BA_total_) values were not calculated either for those groups of vegetables, whenever 100% of the samples had indicated the PHE concentrations <LOD (see Table 3). All the BA_total_ and BC_EDTA_ values were <1, indicating that none of the analyzed groups of vegetables had potential for PHE accumulation (Figure 2a,c). However, the BC_F1_ values were >1, indicating the potential of Tl accumulation in roots and leaves, Sb in tubers, and Cu in seeds (Figure 2b). The values of the calculated BA_total_ coefficient indicated the obvious trend of the Cd, Hg, and Tl accumulation in roots and leaves, with Cd and Hg in tubers, and Hg in legumes. The one-way ANOVA analysis also revealed the significant differences of bioaccumulation coefficient (BA) values for investigated PHEs in different plants parts (Table S3). Calculated BA values were significantly different: for Cu in seed than in other investigated parts of vegetables; for Pb in legume than in other investigated parts of vegetables; and for Sb in tuber than in other investigated parts of vegetables. Moreover, considering investigated regions calculated BA values were significantly different (Table S4): For Cd in Śląskie than in Małopolskie, Świętokrzyskie and Opolskie regions; for Co in Małopolskie than in other regions; for Sb in Świętokrzyskie than in Opolskie and Śląskie regions; and for Zn in Opolskie than in other regions.

The bioconcentration coefficient (BC_F1_ and BC_EDTA_) values, calculated based on the available portions of PHEs in soils, indicated root capacity to adsorb Tl, tuber capacity to adsorb Sb, leaf capacity to adsorb Tl, Zn, and Cd, legume, and seed capacity to adsorb Cu, Sb, and Zn. The result of one-way ANOVA tests revealed that calculated BC_F1_ values were significantly different (Table S5): for Cd in leaf than in fruit and seed; for Cu in seed than in other investigated parts of vegetables; for Pb in legumes than in other investigated parts of vegetables; for Sb in tuber than in other investigated parts of vegetables; and for Zn in seed than in other investigated parts of vegetables. Considering investigated regions calculated BC_F1_ values were significantly different (Table S6): for Cd in Małopolskie than in Śląskie and Opolskie regions; for Co in Małopolskie than in other regions; for Pb in Małopolskie than in Śląskie region; for Sb in Małopolskie and Opolskie than in Świętokrzyskie and Śląskie regions; and for Zn in Świętokrzyskie than in Śląskie and Opolskie regions.

For calculated BC_EDTA_ values the one-way ANOVA analysis revealed significant differences (Table S7) for Cd in leaf than in fruit and seed and for Pb in legume than in other investigated parts of vegetables. Considering investigated regions calculated BC_EDTA_ values were significantly different (Table S8): for Cd in Świętokrzyskie and Małopolskie than in Śląskie and Opolskie regions; for Pb in Małopolskie than in Śląskie, Opolskie and Świętokrzyskie regions; and for Zn in Świętokrzyskie than in Opolskie and Śląskie regions. The results of Jolly et al. [77] revealed that the soil-to-plant transfer indices differed significantly between locations and plant species and those might be related to soil nutrient management and soil properties. The bioconcentration of Cd and Zn in vegetables could be explained by high contents of these elements in acid-soluble fraction (F1 step in the BCR extraction procedure) i.e., Cd 45%–59% and Zn 10%–60% of the total content in soil [22]. Despite acid-soluble content (F1 BCR) were relatively not high for Cu, Tl and Sb (up to 5% of the total concentration in soil) [22] BC values were the highest for these elements. Investigations of other researchers pointed that the highest values of bioaccumulation factors were stated for leafy [47,53,78] or root [79,80] vegetables, with reservations that many other factors should be considered in determining PHEs transfer from soil to plants, as reported by [54,56,57,58,77]. Moreover, heterogeneous accumulation of PHEs in different vegetable species, and different plant parts of same vegetable species could be attributed to their diverse morphological characteristics and position of edible plant parts in respect of their distance from roots and selective uptake of PHEs by each plant [47]. Thus, further investigations in this research area are being conducted. It is also worth of mentioning here, that investigated vegetables might be classified to different parts of plants by various researchers. 

### 3.4. Human Health Risk Assessment

Edible plant cultivation on soils with elevated PHE contents causes presents of these elements in plants tissues. The prolonged consumption of PHEs in foodstuffs may lead to the disruption of numerous biological and biochemical processes in humans [47]. Vegetable consumption is the main source of PHEs introducing to humans [58]. Moreover, BCF values calculated for Tl, Sb, and Cu were >1, what indicated that these elements were cumulated in investigated plants. Thus, human health risk assessment calculations become the crucial part of the research.

#### 3.4.1. Daily Intake Rates

Daily consumed amounts of specific groups of food are crucial in determination of the intake rate of contaminants that can be potentially present in meals eaten. The calculated daily intake rate (DIR) values of the consumed PHEs, among the eight analyzed groups of vegetables, were compared with the tolerable daily intakes of trace elements recommended by JECFA (Joint FAO/WHO Expert Committee on Food Additives), World Health Organization (WHO), and the United States Environmental Protection Agency (USEPA) guidelines. Using the provisional maximum tolerable daily intakes (PMTDI), the following values were adopted in this study (mg/kg bw-d): As 0.0021 [81], Cd 0.0008 [82], Co 0.0014 [83], Cu 0.5 [84], Hg 0.0006 [85], Ni 0.005 [82], Sb 0.006 [86], Tl 0.00014 [87], and Zn 1 [84]. 

The PHE intake rates of the consumed vegetables were presented as a percentage of PMTDI (%PMTDI) (Figure S3). The general trend of increasing PHE intake rates in the consumed vegetables was as follows: adults, according to the Polish statistical data (PL) < adults, according to the USEPA recommended values of consumption < children, according to the USEPA recommended values of consumption. Considering the mean PHE concentrations for adults (under the PL consumption scenario), the highest intake of all the PHEs, except for Tl, was established for tuber vegetables. That results from the fact that potato consumption is the highest among tuber vegetables (100 kg/person/year) in Poland and twice as high in comparison to that of Western European countries [88]. However, the consumption of unnecessary PHEs, as a percentage of PMTDI, was as follows: Cd 23%, Hg 5.0%, Ni 3.1%, Pb 2.6%, and As 0.4%. For Tl, the highest consumption, as a percentage of PMTDI, was determined in root (13.0%), followed by tuber (7.4%) and leafy (5%) vegetable consumption. Considering the mean PHE contents in both adults and children, according to the USEPA recommended values of consumption, the highest intake of unnecessary PHEs was established in root vegetables, followed by tuber and leafy ones (also in legume vegetables for Hg). The mean PHE contents of essential elements were determined in root, legumes, and seed vegetables. The intake values of unnecessary PHEs, as a percentage of PMTDI, by adults and children (under the USEPA consumption scenario) were equal to: As 1.0% and 2.5%, Cd 14% and 33%, Hg 1.7% and 3.9%, Ni 2.9% and 6.6%, Sb 2.4% and 5.5%, and Tl 43% and 100%, respectively. 

The contribution of different groups of vegetables to the PHE daily intakes under the analyzed consumption scenarios was presented in Figure S4. The highest intake for all the analyzed PHEs was observed in root, tuber and leafy vegetables, while in case of essential elements also in seed, legume, and fruit vegetables. Considering the vegetable consumption by adult Poles, it was observed that the main intake was via tuber vegetables (as mentioned before in reference to potatoes mainly), in reference to both essential and unnecessary PHEs. The highest value was determined for Sb (94% of the daily intake rate from vegetable consumption only) and the lowest for Tl (29%). According to intake rates recommended by USEPA for both adults and children, it was observed that the rates were the highest in root vegetables. In adults, the lowest values were obtained for Cu (17% of daily intake rate from vegetable consumption), while the highest ones for As and Ni (95%). In children, those values were rather comparable: the lowest for Cu (18%) and the highest for Ni (97%), followed by As (91%).

#### 3.4.2. Non-Carcinogenic Risk of PHEs

**Figure 3:**
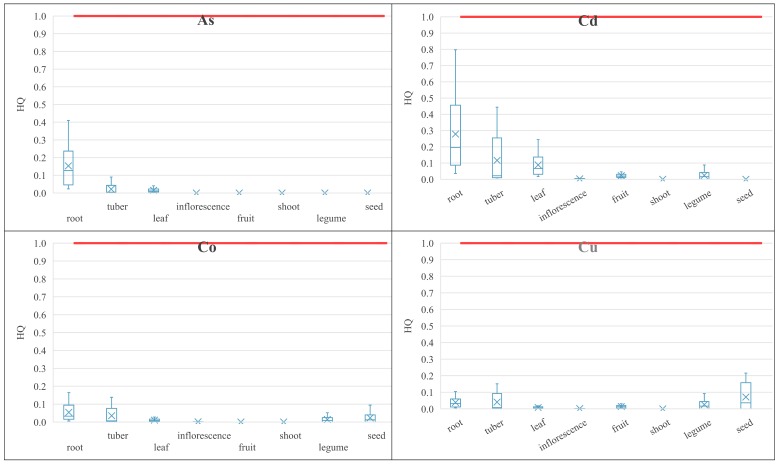

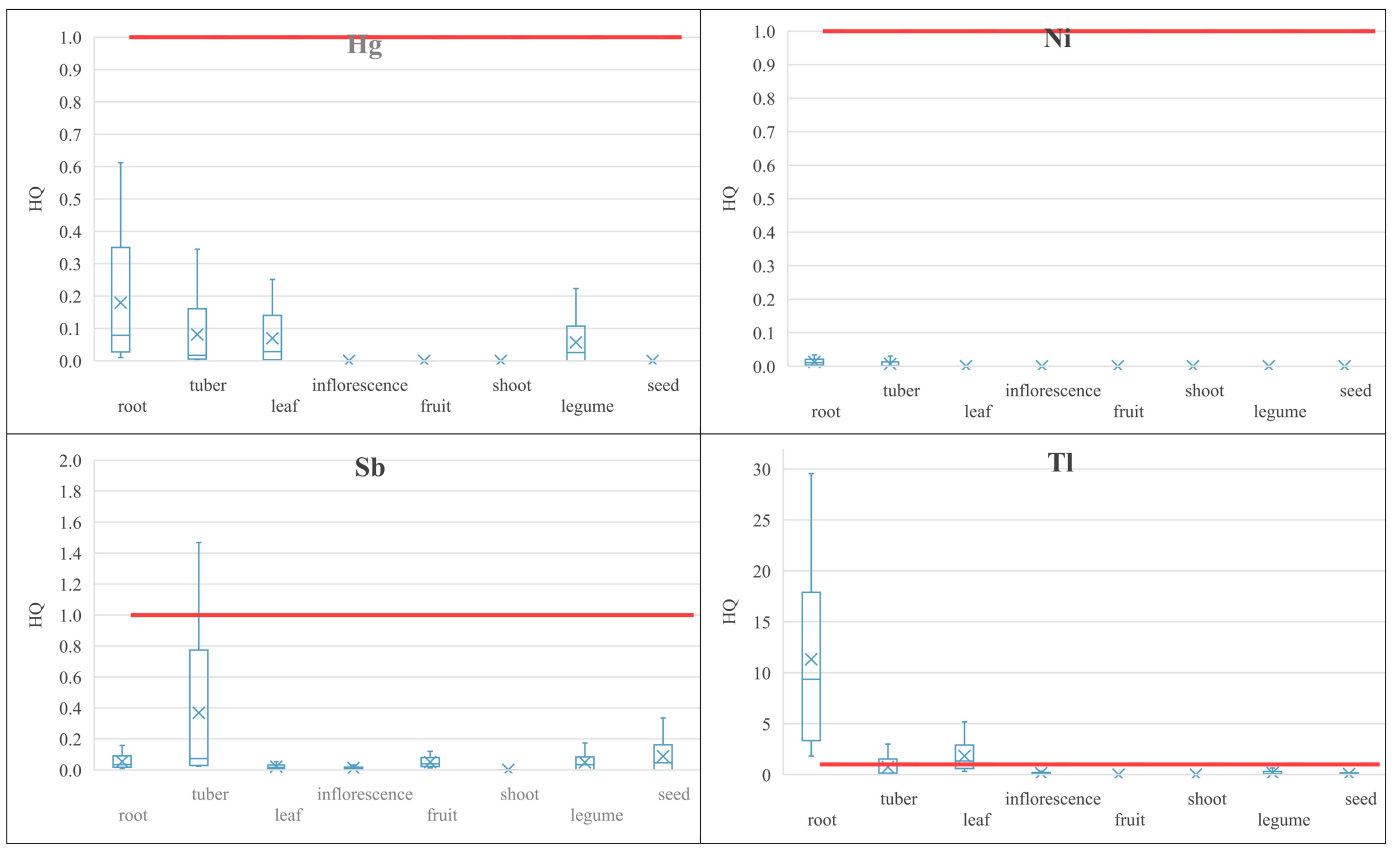

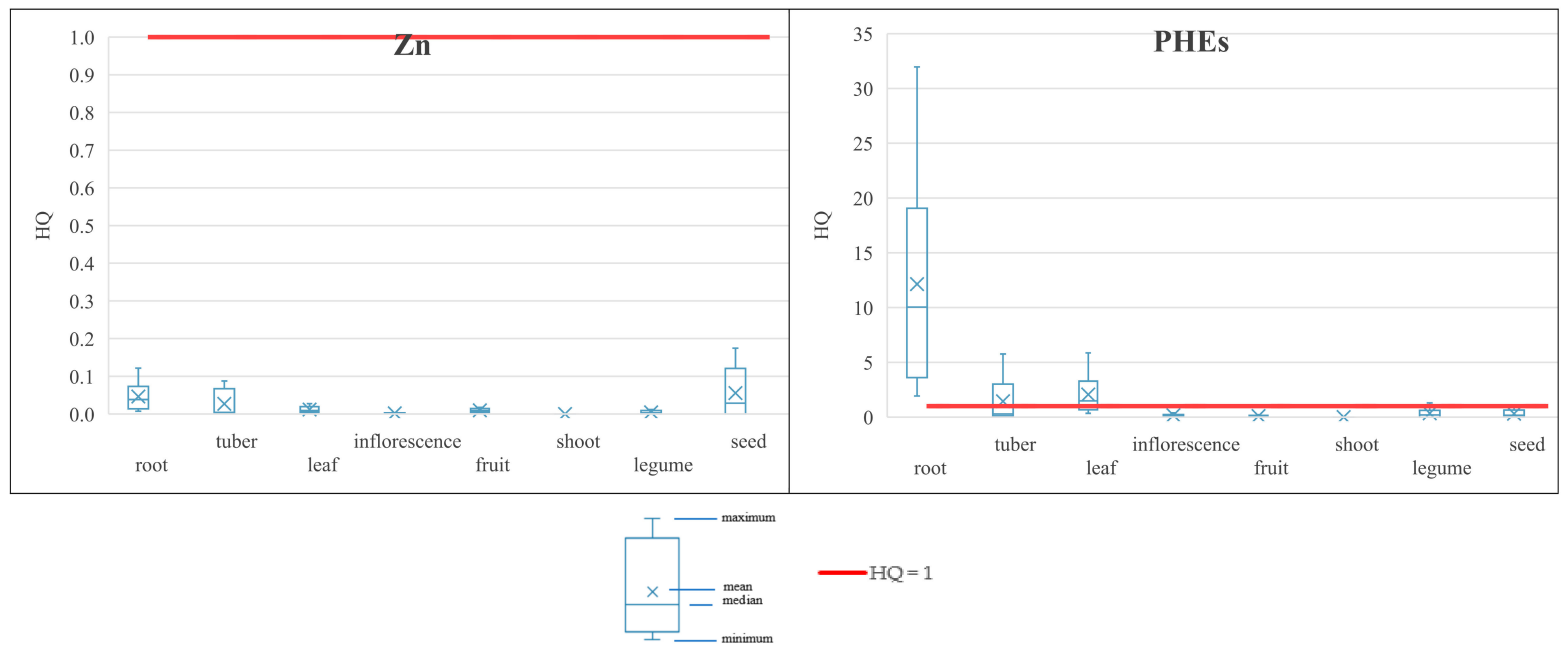
The HQ values of PHEs caused by vegetable ingestion.

The target non-carcinogenic risk value described as hazard quotient (HQ) was set to be equal to 1, according to USEPA [30], as well as the Polish Regulation of the Minister of the Environment on the conduct of the assessment of contamination of the surface of the earth [89]. Statistical characterization of the calculated HQ values for the respective groups of vegetables was presented in Figure 3 under the analyzed intake scenarios. The target HQ = 1 value was exceeded for particular PHEs in the case of Sb in tuber vegetables and Tl in root, leafy, and tuber vegetables. For other PHEs, the highest HQ values were observed in root, tuber, and leafy vegetables in reference to unnecessary elements, except of Hg for which elevated HQ values occurred also in legumes. The total non-carcinogenic risk in particular groups of vegetables (calculated as the sum of HQ values of single PHEs) revealed that the target non-carcinogenic risk values were significantly exceeded in root vegetables (mean HQ = 12.1) under the assessed vegetable intake scenarios, and also exceeded for leafy (mean HQ = 2.1) and tuber (mean HQ = 1.4) vegetables.

#### 3.4.3. Carcinogenic Risk of PHEs

The acceptable carcinogenic risk level was set to be equal to 1 × 10^−5^, as defined in the Polish Regulation of the Minister of the Environment on the conduct of the assessment of contamination of the surface of the earth [89]. In this study, only As was investigated as carcinogenic, according to the current knowledge of the SF values for chemical substances.

**Figure 4:**
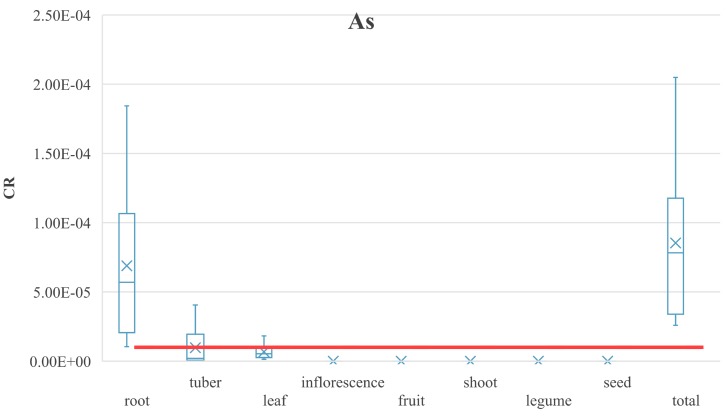
Carcinogenic risk (CR) values for As caused by vegetable ingestion.

The statistical characterization of the calculated CR values for the respective groups of vegetables was presented in Figure 4 under the analyzed intake scenarios. The acceptable CR level of As was exceeded in root vegetables in all the investigated groups of vegetables. Considering the mean CR value, the level was equal to 1.72 × 10^−5^ under the PL adult intake scenario; 5.69 × 10^−5^ under the USEPA adult intake scenario, and 1.33 × 10^−4^ under the USEPA child intake scenario. In the case of tuber vegetables, the acceptable carcinogenic risk level was exceeded, since the CR value was equal to 2.65 × 10^−5^ under the PL adult intake scenario. The acceptable carcinogenic risk levels were not exceeded in either adults or children under the USEPA scenario: the calculated CR values were equal to 1.15 × 10^−6^ and 1.47 × 10^−6^, respectively. In the case of leafy vegetables, the mean CR values were exceeded under the USEPA children intake scenario, since the value was equal to 1.28 × 10^−5^. The acceptable carcinogenic risk level was not exceeded in adults: CR was equal to 5.27 × 10^−6^ under the PL adult scenario and 2.40 × 10^−6^ under the USEPA scenario. The total carcinogenic risk of As (calculated as the sum of CR values for specific groups of vegetables) revealed the unacceptable risk: The mean total CR value was equal to 8.54 × 10^−5^ under the investigated scenarios.

#### 3.4.4. Margin of Exposure to Pb

The BMDL values are used to determine health risk from the exposure to Pb, although those values are not health-based guidance values but rather the levels below which health risk is considered to be acceptable low [29]. Thus, using the MOE approach, the values <1 indicate high health risk, while the values of MOE > 1 point at acceptable low risk. Considering the exposure scenario for PL adults, via the total daily vegetable consumption, the MOE value was equal to 3.1 for Pb. The MOE value was equal to 7.7 for Pb in adults under the USEPA daily vegetable intake scenario, and to 1.6 (Table 5) in children. At the P95 exposure level, the MOE values of Pb in adult Poles and children under the USEPA scenario were equal to 0.9 and 0.4, respectively, while the MOE value was equal to 2.2 in adults under the USEPA intake scenario. Under all the three investigated exposure scenarios, at mean exposure levels, as well as at the P95 exposure scenario in USEPA adults, the calculated MOE values were >1, indicating low health risk of the Pb intake from the daily consumption of vegetables. At the P95 exposure level, the calculated MOE values in adults, under the Polish statistical data exposure scenario, and in children, under the USEPA exposure scenario, the calculated MOE values were <1, indicating high risk of the Pb intake via the daily consumption of vegetables.

#### 3.4.5. Uncertainties in HHRA

The values of calculated risk depended strongly on the assumed intake rates. The statistical data, averaged for a large general population, were the values applied in this study. The Polish statistical data concerning intake rates were available only for adults and concerned only the most commonly consumed groups of products in Poland. The intake rate of vegetables equal to 389 g was so high due to the consumption of potatoes that was twice as high in comparison to the Western European countries. Thus, also the recommended consumed amounts of vegetables from the USEPA guidelines were used for the HHRA calculations. Unfortunately, no statistical data were available for children in Poland, thus, also in that case, the recommended values of vegetable consumption by children were used in the HHRA analysis. The amount of daily consumed vegetables, according to the values recommended by USEPA, was equal to 227 g in adults and 121 g in children. Moreover, there were differences in the assignment of particular vegetables to their groups in respect of the edible parts of plants among various studies, and that could also have some impact on the calculated intake rates and indirectly on risk values. It was evidenced that the local statistical data, obtained exactly on site, were necessary for the reliable risk value assessment. In addition, risk assessment required the values of some parameters that were normally not investigated under general statistical research projects. The contributions of various groups of consumed vegetables in the daily PHE intakes were based on the assumption that vegetable consumption constituted 100% of the PHE intake, meaning that other PHE ingestion sources were not taken into consideration (besides inhalation and dermal exposure). Thus, if fruit and cereals were included as other groups of consumed plants (again omitting other food product groups, i.e., meat, dairy, and processed food products), the risk values would be different than the calculated values.

## 4. Conclusions

The present studies investigated concentrations of PHEs in eight groups of vegetables cultivated on the arable soils of southern Poland, and, in reference to that, the human health-risk implications were considered as well. Although PHE concentrations in arable soils of the specific Polish regions were below permissible levels [22], the mean contents of Hg in roots, leaves, and legumes, determined in the current research, as well as the Cd concentrations in roots, were on the border of the maximum allowable concentrations. The contents of other PHEs in the investigated parts of vegetables were not close to the limit values, specified in the European Commission regulation on maximum levels for certain contaminants in foodstuff. The HHRA analysis was calculated basing on the statistical amounts of consumed edible plants. The target non-carcinogenic risk to human health from the total PHEs content in the analyzed vegetables was exceeded in root, as well as in leafy and tuber vegetables. The total carcinogenic risk of As for specific groups of vegetables was assessed as unacceptable risk. The risk from Pb intake via vegetables, according to the MOE approach, might occur at the P95 exposure level, in adults, under the Polish statistical data exposure scenario, and in children, under the USEPA exposure scenario. It was proved that the HHRA analysis was recommended, in the light of the latest scientific findings concerning toxicological properties of chemicals, even where the environmental quality standards were maintained. However, precise local statistical data were required to achieve the real risk values.

## Figures and Tables

**Table 1:** Description of the vegetables investigated in this study.

No.	Common Name	Botanical Name	Number of Samples (*n*)	Edible Part
1.	Beet root	Beta vulgaris	11	root
2.	Carrot	Daucus carota	7	root
3.	Celery root	Apium graveolens	8	root
4.	Horseradish	Armoracia rusticana	4	root
5.	Parsley root	Petroselinum hortense	5	root
6.	Radish	Raphanus sativus var. sativus	4	root
7.	Turnip	Brassica rapa	2	root
8.	Garlic	Allium sativum	5	tuber
9.	Kohlrabi	Brassica oleracea var. gongylodes	8	tuber
10.	Onion	Allium cepa	9	tuber
11.	Potato	Solanum tuberosum	5	tuber
12.	Arugula	Eruca sativa	3	leaf
13.	Beet tops	Beta vulgaris	8	leaf
14.	Cabbage	Brassica oleracea var. capitata f. alba	5	leaf
15.	Celery tops	Apium graveolens	7	leaf
16.	Chives	Allium schoenoprasum	9	leaf
17.	Kale	Brassica oleracea var. sabellica	3	leaf
18.	Leek	Allium porrum	6	leaf
19.	Lettuce	Lactuca sativa	8	leaf
20.	Lovage	Levisticum officinale	4	leaf
21.	Parsley tops	Petroselinum hortense	8	leaf
22.	Rhubarb	Rheum rhaponticum	8	leaf
23.	Spinach	Spinacia oleracea	5	leaf
24.	Broccoli	Brassica oleracea	5	inflorescence
25.	Cauliflower	Brassica oleracea var. botrytis	5	inflorescence
26.	Zucchini	Cucurbita pepo convar. giromontiina Greb	6	fruit
27.	Pumpkin	Cucurbita pepo	4	fruit
28.	Cucumber	Cucumis sativus	6	fruit
29.	Sweet pepper	Capsicum annuum	4	fruit
30.	Tomato	Lycopersicon esculentum	6	fruit
31.	Asparagus	Asparagus officinalis	2	shoot
32.	Green bean	Phaseolus vulgaris	7	legume
33.	Broad bean	Vicia faba	5	seed
34.	Kidney bean	Phaseolus vulgaris	4	seed
35.	Pea	Pisum sativum	4	seed
36.	Pumpkin seeds	Cucurbita pepo	6	seed

**Table 2:** Intake rates (IR) (g ww./person-day) of the consumed groups of vegetables.

IR (g ww./Person-Day)			
Vegetable Part	Adult PL	Adult USEPA	Child USEPA
root	23.3	77	38.4
tuber	289.5	12.6	3.45
leaf	15.6	7.1	8.1
inflorescence	5.75	6.3	3.3
fruit	45	84.7	32.7
shoot	nd	1.4	0.3
legume	nd	14	9.75
seed	nd	23.8	24.6

nd—not defined.

**Table 3:** Concentrations of potentially harmful elements (PHEs) (mg/kg ww.) in the groups of vegetables.

PHE	Statistical Parameters mg/kg ww.	Root (*n* = 41)	Tuber (*n* = 27)	Leaf (*n* = 74)	Inflorescence (*n* = 10)	Fruit (*n* = 29)	Shoot (*n* = 2)	Legume (*n* = 7)	Seed (*n* = 19)
As	min	nd	nd	nd	ND	ND	ND	ND	ND
	mean ± SD	0.021 ± 0.004	0.002 ± 0.001	0.009 ± 0.006					
	max	0.056	0.009	0.028					
	P95	0.048	0.007	0.023					
	>LOD (%)	27.2	12.7	25.6	0.00	0.00	0.00	0.00	0.00
Cd	min	nd	nd	nd	nd	nd	ND	nd	ND
	mean ± SD	0.107 ± 0.012	0.046 ± 0.014	0.162 ± 0.016	0.017 ± 0.008	0.012 ± 0.005		0.038 ± 0.005	
	max	0.375	0.119	0.556	0.031	0.022		0.144	
	P95	0.311	0.107	0.453	0.029	0.021		0.135	
	>LOD (%)	88.6	86.0	97.6	90.0	80.2	0.00	71.4	0.00
	MAC	0.10	0.10	0.20	0.05	0.05	0.05	0.05	0.05
Co	min	nd	nd	nd	nd	ND	ND	nd	nd
	mean ± SD	0.005 ± 0.003	0.004 ± 0.003	0.007 ± 0.002	0.002 ± 0.001			0.006 ± 0.002	0.004 ± 0.002
	max	0.029	0.012	0.017	0.005			0.027	0.022
	P95	0.019	0.010	0.015	0.004			0.024	0.017
	>LOD (%)	15.0	30.0	46.0	30.0	0.00	0.00	57.1	36.7
Cu	min	nd	0.126	nd	0.151	0.042	0.234	0.128	0.299
	mean ± SD	0.698 ± 0.077	0.712 ± 0.064	0.744 ± 0.068	0.339 ± 0.059	0.303 ± 0.085	0.282 ± 0.014	1.582 ± 0.072	3.373 ± 0.163
	max	1.894	1.768	1.711	0.650	0.620	0.330	7.638	5.403
	P95	1.630	1.462	1.383	0.591	0.574	0.282	5.692	5.264
	>LOD (%)	92.1	100.0	95.8	100.0	100.0	100.0	100.0	100.0
Hg	min	nd	nd	nd	ND	ND	ND	nd	ND
	mean ± SD	0.019 ± 0.009	0.009 ± 0.007	0.018 ± 0.005				0.021 ±0.018	
	max	0.101	0.029	0.163				0.147	
	P95	0.072	0.025	0.140				0.103	
	>LOD (%)	36.4	31.7	44.7	0.00	0.00	0.00	14.3	0.00
	MAC	0.02	0.02	0.02	0.02	0.02	0.02	0.02	0.02
Ni	min	0.073	nd	ND	ND	ND	ND	ND	ND
	mean ± SD	0.130 ± 0.056	0.038 ± 0.022						
	max	0.299	0.186						
	P95	0.264	0.149						
	>LOD (%)	4.20	10.5	0.00	0.00	0.00	0.00	0.00	0.00
Pb	min	nd	nd	0.001	ND	nd	nd	nd	nd
	mean ± SD	0.063 ± 0.011	0.079 ± 0.010	0.094 ± 0.013		0.030 ± 0.008	0.036 ± 0.010	0.093 ± 0.006	0.019 ± 0.009
	max	0.333	0.312	0.340		0.087	0.041	0.580	0.070
	P95	0.248	0.262	0.278		0.079	0.036	0.420	0.059
	>LOD (%)	38.0	43.2	69.2	0.00	40.2	50.0	57.1	28.7
	MAC	0.10	0.10	0.30	0.10	0.10	0.10	0.10	0.10
Sb	min	nd	nd	nd	nd	nd	nd	nd	nd
	mean ± SD	0.007 ± 0.004	0.053 ± 0.012	0.018 ± 0.004	0.015 ± 0.008	0.008 ± 0.002	0.013 ± 0.006	0.031 ± 0.004	0.026 ± 0.006
	max	0.071	0.163	0.086	0.071	0.083	0.002	0.116	0.097
	P95	0.068	0.142	0.081	0.058	0.081	0.013	0.107	0.082
	>LOD (%)	38.8	44.5	55.5	40.0	31.6	50.0	42.9	30.0
Tl	min	nd	nd	nd	nd	ND	ND	nd	nd
	mean ± SD	0.055 ± 0.001	0.003 ± 0.001	0.031 ± 0.002	0.008 ± 0.002			0.002 ± 0.001	0.001 ± 0.000
	max	0.452	0.008	0.115	0.017			0.014	0.002
	P95	0.408	0.007	0.096	0.015			0.010	0.001
	>LOD (%)	43.1	38.5	53.7	60.0	0.00	0.00	28.6	12.5
Zn	min	2.22	2.50	4.51	2.64	1.23	3.93	2.52	10.5
	mean ± SD	6.72 ± 1.24	4.36 ± 0.91	9.46 ± 1.25	3.72 ± 1.25	1.85 ± 0.75	4.89 ± 1.08	3.94 ± 0.90	18.8 ± 1.29
	max	16.2	6.77	16.4	5.05	2.58	5.86	5.64	34.9
	P95	14.2	6.36	15.3	4.88	2.46	4.89	5.46	32.0
	>LOD (%)	100.0	100.0	100.0	100.0	100.0	100.0	100.0	100.0

ww. wet weight; P95: 95th percentile; nd: <LOD; ND: <LOD in all analyzed samples; MAC: maximum allowable concentration [73]; LOD: limit of detection.

**Table 4:** Factor loading results obtained from the principal components analysis (PCA).

PHEs	Varimax Rotated			
	PC1	PC2	PC3	PC4
As	**0.716**	0.112	**0.586**	0.041
Cd	**0.646**	0.106	−0.055	**0.512**
Co	**0.871**	0.251	−0.129	0.084
Cu	0.095	**0.972**	0.000	0.010
Hg	**0.926**	0.021	0.102	0.094
Ni	−0.103	0.012	**0.902**	−0.042
Pb	**0.558**	0.161	0.154	**0.656**
Sb	0.022	0.109	−0.011	**0.908**
Tl	**0.594**	0.237	**0.639**	0.249
Zn	0.197	**0.907**	0.149	0.267
Eigenvalues	4.57	1.52	1.41	1.02
Explained variance %	32.7	19.5	16.4	16.7
Cumulative variance %	32.7	52.2	68.6	85.3

Factor loadings exceeding 0.5 are shown in bold.

**Table 5:** Margin of exposure (MOE) values for Pb caused by vegetable ingestion.

Vegetable Intake Scenario	MOE (Mean Exposure)	MOE (P95 Exposure)
Adult PL	3.1	0.9
Adult USEPA	7.7	2.2
Child USEPA	1.6	0.4

P95 95th percentile. PL—Polish statistical data, USEPA—United States Environmental Protection Agency.

## Supplementary Materials

The following are available online at https://www.mdpi.com/1660-4601/16/20/4053/s1, Figure S1: Dendogram of PHEs in vegetable samples according to Sneath’s criteria; Figure S2: The color-scale map representing standardized contents of PHEs in vegetables; Figure S3: Daily intake rates of PHEs via consumed vegetables, as a percentage of provisional maximum tolerable daily intake (%PMTDI); Figure S4: The contribution of various groups of vegetables to the PHE daily intake rates; Table S1: Results of one-way ANOVA of differences between average concentrations of PHEs in groups of vegetables; Table S2: Results of one-way ANOVA of differences between average concentrations of PHEs in investigated regions of southern Poland; Table S3: Results of one-way ANOVA of differences between average BA_total_ values of PHEs in groups of vegetables; Table S4: Results of one-way ANOVA of differences between BA_total_ values of PHEs in investigated regions of southern Poland; Table S5: Results of one-way ANOVA of differences between average BC_F1_ values of PHEs in groups of vegetables; Table S6: Results of one-way ANOVA of differences between BC_F1_ values of PHEs in investigated regions of southern Poland; Table S7: Results of one-way ANOVA of differences between average BC_EDTA_ values of PHEs in groups of vegetables; Table S8: Results of one-way ANOVA of differences between BC_EDTA_ values of PHEs in investigated regions of southern Poland.
