# Supplementary material for: Potentially Harmful Element Concentrations in the Vegetables Cultivated on Arable Soils, with Human Health-Risk Implications
Source: Int J Environ Res Public Health. 2019 Oct 22;16(20):4053. doi: 10.3390/ijerph16204053 (PMC6843946; doi:10.3390/ijerph16204053)
Supplement: Supplementary file 1 [file ijerph-16-04053-s001.pdf]

International Journal of
Environmental Research and Public Health

Supplementary material

Potentially Harmful Element concentrations in the vegetables cultivated on arable soils, with human health-risk implications

Agnieszka Gruszecka-Kosowska [1*]

[1] AGH University of Science and Technology, Faculty of Geology, Geophysics, and Environmental Protection, Department of Environmental Protection, Al. Mickiewicza 30, 30-059 Kraków, Poland; agnieszka.gruszecka@agh.edu.pl
ORCID 0000-0002-4988-173X

* Correspondence: agnieszka.gruszecka@agh.edu.pl (A.G-K.)

Table S1. Results of one-way ANOVA of differences between average concentrations of PHEs in groups of vegetables.

Figure S1. Dendogram of PHEs in vegetable samples according to Sneath's criteria.

Figure S2. The color-scale map representing standardized contents of PHEs in vegetables.

Table S2. Results of one-way ANOVA of differences between average concentrations of PHEs in investigated regions of southern Poland.

Table S3. Results of one-way ANOVA of differences between average BA_{total} values of PHEs in groups of vegetables.

Table S4. Results of one-way ANOVA of differences between BA_{total} values of PHEs in investigated regions of southern Poland.

Table S5. Results of one-way ANOVA of differences between average BC_{F1} values of PHEs in groups of vegetables.

Table S6. Results of one-way ANOVA of differences between BC_{F1} values of PHEs in investigated regions of southern Poland.

Table S7. Results of one-way ANOVA of differences between average BC_{EDTA} values of PHEs in groups of vegetables.

Table S8. Results of one-way ANOVA of differences between BC_{EDTA} values of PHEs in investigated regions of southern Poland.

Figure S3. Daily intake rates of PHEs via consumed vegetables, as a percentage of provisional maximum tolerable daily intake (%PMTDI).

Figure S4. The contribution of various groups of vegetables to the PHE daily intake rates.

Table S1. Results of one-way ANOVA of differences between average concentrations of PHEs in groups of vegetables.

PHE	F	p	Confidence interval	
**As**	0.9528	0.4834	0.95	Non-significant differences
**Cd**	1.4546	0.2238	0.95	Non-significant differences
**Co**	0.4921	0.8321	0.95	Non-significant differences
**Cu**	1.3574	0.2617	0.95	Non-significant differences
**Hg**	0.5794	0.7666	0.95	Non-significant differences
**Ni**	1.1671	0.3525	0.95	Non-significant differences
**Pb**	0.7209	0.6554	0.95	Non-significant differences
**Sb**	1.0261	0.4352	0.95	Non-significant differences
**Tl**	0.5313	0.8033	0.95	Non-significant differences
**Zn**	0.8989	0.5210	0.95	Non-significant differences

F – F-ratio
p – probability

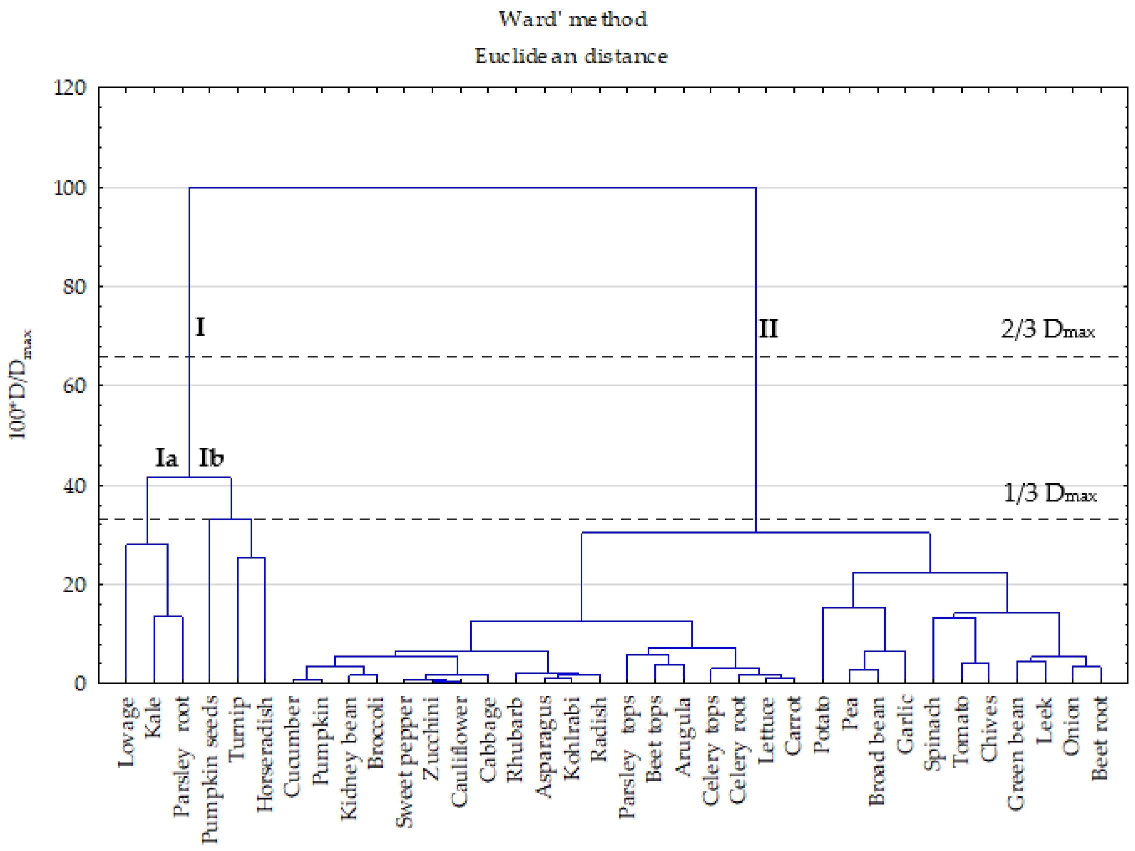

Figure S1. Dendogram of PHEs in vegetable samples according to Sneath's criteria

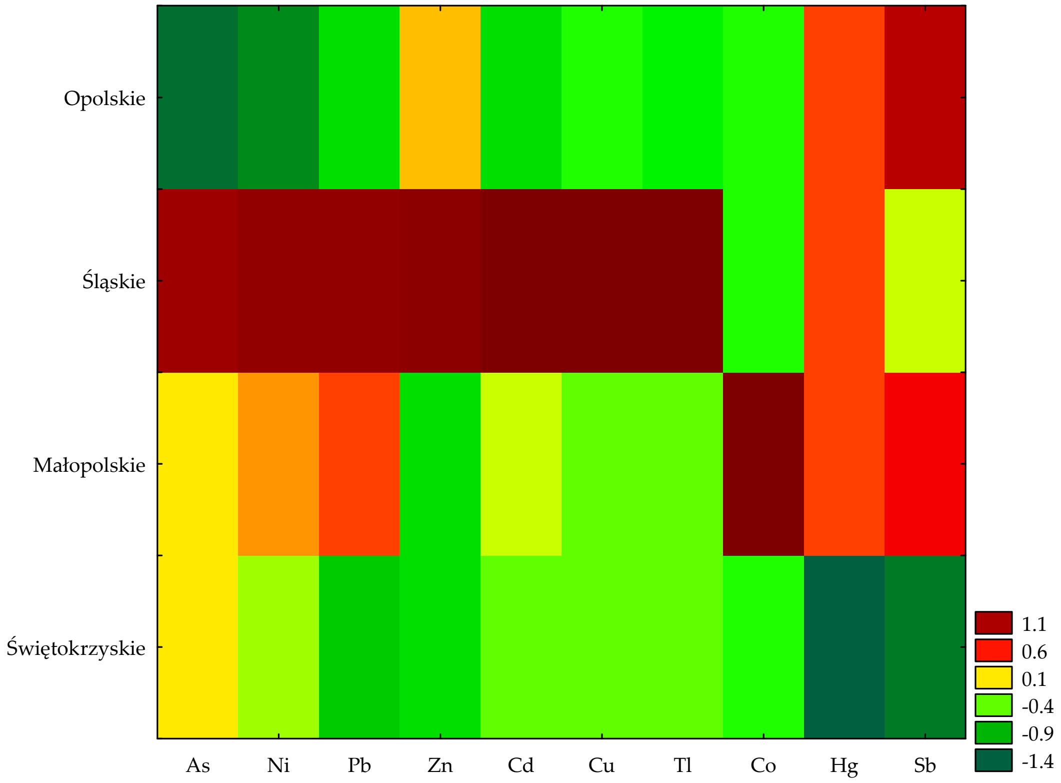

Figure S2. The color-scale map representing standardized contents of PHEs in vegetables.

Table S2. Results of one-way ANOVA of differences between average concentrations of PHEs in investigated regions of southern Poland.

PHE	F	p	Confidence interval	Fisher's LSD test, probabilities for post-hoc tests				
**As**	0.4209	0.7387	0.95	Non-significant differences				
**Cd**	6.1892	**0.00094**	0.95	Error: between MS=0.0662, df=63.000				
					{1}	{2}	{3}	{4}
					0.0158	0.3787	0.0892	0.0574
				Opolskie		**0.0136**	0.5971	0.7689
				Śląskie	**0.0136**		**0.0006**	**0.0003**
				Małopolskie	0.5971	**0.0006**		0.6789
				Świętokrzyskie	0.7689	**0.0003**	0.6789	
**Co**	6.5004	**0.00067**	0.95	Error: between MS=0.00011, df=63.000				
					{1}	{2}	{3}	{4}
					0.0068	0.0008	0.0126	0.0005
				Opolskie		0.3102	0.3115	0.2848
				Śląskie	0.3102		**0.0007**	0.9463
				Małopolskie	0.3115	**0.0007**		**0.0003**
				Świętokrzyskie	0.2848	0.9463	**0.0003**	
**Cu**	2.5365	0.0646	0.95	Non-significant differences				
**Hg**	1.8195	0.1527	0.95	Non- significant differences				
**Ni**	0.5581	0.6447	0.95	Non- significant differences				
**Pb**	2.0414	0.1171	0.95	Non- significant differences				
**Sb**	1.6992	0.1762	0.95	Non- significant differences				
**Tl**	0.8403	0.4769	0.95	Non- significant differences				
**Zn**	3.0565	**0.0347**	0.95	Error: between MS=246.14, df=63.000				
					{1}	{2}	{3}	{4}
					32.860	9.3709	8.3052	7.9311
				Opolskie		**0.0090**	**0.0049**	**0.0051**
				Śląskie	**0.0090**		0.8283	0.7818
				Małopolskie	**0.0049**	0.8283		0.9364
				Świętokrzyskie	**0.0051**	0.7818	0.9364	

F – F-ratio
p – probability
values <0.05 are shown in **bold**

Table S3. Results of one-way ANOVA of differences between average BA_{total} values of PHEs in groups of vegetables.

PHE	F	p	Confidence interval	Fisher's LSD test, probabilities for post-hoc tests								
**As**	0.7886	0.5980	0.95	Non-significant differences								
**Cd**	1.8473	0.0831	0.95	Non-significant differences								
**Co**	1.8195	0.0883	0.95	Non-significant differences								
**Cu**	3.3332	**0.0026**	0.95	Error: between MS=0.0150, df=136.000								
					{1}	{2}	{3}	{4}	{5}	{6}	{7}	{8}
					0.0227	0.0269	0.0184	0.0129	0.0120	0.0061	0.0471	0.1751
				root		0.9123	0.8817	0.8418	0.7660	0.8007	0.7096	0.0001
				tuber	0.9123		0.8087	0.7914	0.7168	0.7618	0.7685	0.0008
				leaf	0.8817	0.8087		0.9069	0.8458	0.8483	0.6524	0.0000
				inflorescence	0.8418	0.7914	0.9069		0.9864	0.9286	0.6486	0.0027
				fruit	0.7660	0.7168	0.8458	0.9864		0.9305	0.6014	0.0001
				shoot	0.8007	0.7618	0.8483	0.9286	0.9305		0.6368	0.0149
				legume	0.7096	0.7685	0.6524	0.6486	0.6014	0.6368		0.0640
				seed	**0.0001**	**0.0008**	**0.0000**	**0.0027**	**0.0001**	**0.0149**	0.0640	
**Hg**	1.0444	0.4032	0.95	Non-significant differences								
**Ni**	0.8287	0.5652	0.95	Non-significant differences								
**Pb**	3.7570	**0.00094**	0.95	Error: between MS=0.00000, df = 136.000								
					{1}	{2}	{3}	{4}	{5}	{6}	{7}	{8}
					0.00024	0.00033	0.00058	0.0000	0.00042	0.00020	0.00329	0.00018
				root		0.80688	0.23588	0.62058	0.60228	0.94843	**0.00000**	0.88019
				tuber	0.8069		0.47592	0.52544	0.82069	0.84235	**0.00002**	0.72616
				leaf	0.2359	0.47592		0.20902	0.62550	0.54249	**0.00003**	0.25434
				inflorescence	0.6206	0.52544	0.20902		0.40180	0.78891	**0.00001**	0.72684
				fruit	0.6023	0.82069	0.62550	0.40180		0.73254	**0.00002**	0.55147
				shoot	0.9484	0.84235	0.54249	0.78891	0.73254		**0.00036**	0.98192
				legume	**0.0000**	**0.00002**	**0.00003**	**0.00001**	**0.00002**	**0.00036**		**0.00001**
				seed	0.8802	0.72616	0.25434	0.72684	0.55147	0.98192	**0.00001**	
**Sb**	2/7197	**0.0114**	0.95	Error: between MS=0.00042, df = 136.000								
					{1}	{2}	{3}	{4}	{5}	{6}	{7}	{8}
					0.00584	0.02986	0.00730	0.00972	0.00527	0.00246	0.01195	0.00696

				root		**0.0003**	0.7635	0.6352	0.9250	0.7574	0.5756	0.8603
				tuber	**0.0003**		**0.0002**	**0.0242**	**0.0005**	**0.0176**	0.1186	**0.0019**
				leaf	0.7635	**0.0002**		0.7560	0.7097	0.6494	0.6616	0.9547
				inflorescence	0.6352	**0.0242**	0.7560		0.6028	0.5619	0.8585	0.7551
				fruit	0.9250	**0.0005**	0.7097	0.6028		0.8017	0.5508	0.8053
				shoot	0.7574	**0.0176**	0.6494	0.5619	0.8017		0.5115	0.6936
				legume	0.5756	0.1186	0.6616	0.8585	0.5508	0.5115		0.6623
				seed	0.8603	**0.0019**	0.9547	0.7551	0.8053	0.6936	0.6623	
**Tl**	1.2842	0.2626	0.95	Non-significant differences								
**Zn**	1.3158	0.2474	0.95	Non-significant differences								

F – F-ratio

p – probability

values <0.05 are shown in **bold**

Table S4. Results of one-way ANOVA of differences between BA_{total} values of PHEs in investigated regions of southern Poland.

PHE	F	p	Confidence interval	Fisher's LSD test, probabilities for post-hoc tests				
**As**	1.6745	0.1753	0.95	Non-significant differences				
**Cd**	3.9663	**0.0095**	0.95	Error: between MS=0.01704, df=140.000				
					{1}	{2}	{3}	{4}
					0.01791	0.11980	0.04724	0.04853
				Opolskie		**0.0012**	0.3421	0.3214
				Śląskie	**0.0012**		**0.0197**	**0.0220**
				Małopolskie	0.3421	**0.0197**		0.9667
				Świętokrzyskie	0.3214	**0.0220**	0.9667	
**Co**	5.7498	**0.0010**	0.95	Error: between MS=0.00000, df=140.000				
					{1}	{2}	{3}	{4}
					0.00049	0.00032	0.00150	0.00007
				Opolskie		0.6440	**0.0071**	0.2615
				Śląskie	0.6440		**0.0017**	0.5077
				Małopolskie	**0.0071**	**0.0017**		**0.0002**
				Świętokrzyskie	0.2615	0.5077	**0.0002**	
**Cu**	0.7884	0.5023	0.95	Non-significant differences				
**Hg**	0.8759	0.4553	0.95	Non-significant differences				
**Ni**	1.5516	0.2039	0.95	Non-significant differences				
**Pb**	2.2386	0.0864	0.95	Non-significant differences				
**Sb**	5.7403	**0.00099**	0.95	Error: between MS=0.00041, df=140.00				
					{1}	{2}	{3}	{4}
					0.01762	0.00509	0.01443	0.00019
				Opolskie		**0.0096**	0.5050	**0.0004**
				Śląskie	**0.0096**		0.0524	0.3065
				Małopolskie	0.5050	0.0524		**0.0034**
				Świętokrzyskie	**0.0004**	0.3065	**0.0034**	
**Tl**	1.1052	0.3493	0.95	Non-significant differences				
**Zn**	5.9848	**0.00072**	0.95	Error: between MS=9.6152, df=140.00				
					{1}	{2}	{3}	{4}
					2.5647	0.01519	0.01478	0.08058
				Opolskie		**0.0007**	**0.0006**	**0.0009**
				Śląskie	**0.0007**		0.9996	0.9288
				Małopolskie	**0.0006**	0.9996		0.9284
				Świętokrzyskie	**0.0009**	0.9288	0.9284	

F – F-ratio

p – probability

values <0.05 are shown in **bold**

Table S5. Results of one-way ANOVA of differences between average BC_{F1} values of PHEs in groups of vegetables.

PHE	F	p	Confidence interval	Fisher's LSD test, probabilities for post-hoc tests								
**As**	0.6225	0.7366	0.95	Non-significant differences								
**Cd**	2.5052	**0.0188**	0.95	Error: between MS=0.01181, df=136.000								
					{1}	{2}	{3}	{4}	{5}	{6}	{7}	{8}
					0.08801	0.05192	0.11172	0.02364	0.01614	0.00131	0.03487	0.04478
				root		0.2911	0.3604	0.1418	**0.0255**	0.1378	0.3618	0.2064
				tuber	0.2911		0.0587	0.5488	0.3280	0.4061	0.7794	0.8527
				leaf	0.3604	0.0587		**0.0356**	**0.0012**	0.0529	0.1764	**0.0346**
				inflorescence	0.1418	0.5488	**0.0356**		0.8693	0.7376	0.8662	0.6540
				fruit	**0.0255**	0.3280	**0.0012**	0.8693		0.8035	0.7535	0.4334
				shoot	0.1378	0.4061	0.0529	0.7376	0.8035		0.6629	0.4754
				legume	0.3618	0.7794	0.1764	0.8662	0.7535	0.6629		0.8707
				seed	0.2064	0.8527	**0.0346**	0.6540	0.4334	0.4754	0.8707	
**Co**	1.4743	0.1814	0.95	Non-significant differences								
**Cu**	2.5702	**0.0162**	0.95	Error: between MS=18.751, df=136.000								
					{1}	{2}	{3}	{4}	{5}	{6}	{7}	{8}
					1.4005	1.6490	1.1088	0.6768	0.5144	0.0898	4.5618	5.5605
				root		0.8550	0.7774	0.6775	0.4858	0.5722	0.1742	**0.0026**
				tuber	0.8550		0.6663	0.6050	0.4361	0.5206	0.2309	**0.0117**
				leaf	0.7774	0.6663		0.7943	0.6069	0.6519	0.1278	**0.0005**
				inflorescence	0.6775	0.6050	0.7943		0.9287	0.8251	0.1452	**0.0102**
				fruit	0.4858	0.4361	0.6069	0.9287		0.8582	0.0902	**0.0007**
				shoot	0.5722	0.5206	0.6519	0.8251	0.8582		0.1465	**0.0254**
				legume	0.1742	0.2309	0.1278	0.1452	0.0902	0.1465		0.6806
				seed	**0.0026**	**0.0117**	**0.0005**	**0.0102**	**0.0007**	**0.0254**	0.6806	
**Ni**	1.3397	0.2363	0.95	Non-significant differences								
**Pb**	2.4915	**0.0194**	0.95	Error: between MS=1.1125, df = 136.000								
					{1}	{2}	{3}	{4}	{5}	{6}	{7}	{8}
					0.26604	0.25813	0.58089	0.0000	0.54086	0.13433	2.2936	0.16528
				root		0.9809	0.2115	0.5303	0.3751	0.8156	**0.0005**	0.7610
				tuber	0.9809		0.2910	0.5729	0.4256	0.8340	**0.0007**	0.8038

				leaf	0.2115	0.2910		0.1516	0.8868	0.4173	**0.0022**	0.1745
				inflorescence	0.5303	0.5729	0.1516		0.2224	0.8356	**0.0005**	0.7180
				fruit	0.3751	0.4256	0.8868	0.2224		0.4828	**0.0029**	0.2903
				shoot	0.8156	0.8340	0.4173	0.8356	0.4828		**0.0044**	0.9582
				legume	**0.0005**	**0.0007**	**0.0022**	**0.0005**	**0.0029**	**0.0044**		**0.0004**
				seed	0.7610	0.8038	0.1745	0.7180	0.2903	0.9582	**0.0004**	
**Sb**	2.6558	**0.0132**	0.95	Error: between MS=13.048, df = 136.000								
					{1}	{2}	{3}	{4}	{5}	{6}	{7}	{8}
					0.02977	0.15037	0.03624	0.05051	0.02706	0.01270	0.05978	0.03637
				root		**0.0003**	0.7935	0.6188	0.9290	0.7587	0.5892	0.8394
				tuber	**0.0003**		**0.0002**	**0.0279**	**0.0005**	**0.0190**	0.1206	**0.0023**
				leaf	0.7935	**0.0002**		0.7193	0.7400	0.6635	0.6635	0.9965
				inflorescence	0.6188	**0.0279**	0.7193		0.5898	0.5527	0.8841	0.7534
				fruit	0.9290	**0.0005**	0.7400	0.5898		0.8009	0.5656	0.7894
				shoot	0.7587	**0.0190**	0.6635	0.5527	0.8009		0.5220	0.6838
				legume	0.5892	0.1206	0.6635	0.8841	0.5656	0.5220		0.6870
				seed	0.8394	**0.0023**	0.9965	0.7534	0.7894	0.6838	0.6870	
**Tl**	1.4308	0.1979	0.95	Non-significant differences								
**Zn**	2.1520	**0.0423**	0.95	Error: between MS=0.12709, df = 136.000								
					{1}	{2}	{3}	{4}	{5}	{6}	{7}	{8}
					0.09476	0.11840	0.16816	0.08478	0.04111	0.01310	0.09617	0.44996
				root		0.8327	0.3881	0.9444	0.6081	0.6690	0.9941	**0.0018**
				tuber	0.8327		0.6295	0.8279	0.5191	0.5981	0.9113	**0.0095**
				leaf	0.3881	0.6295		0.5412	0.1828	0.4048	0.6986	**0.0070**
				inflorescence	0.9444	0.8279	0.5412		0.7701	0.7432	0.9584	**0.0194**
				fruit	0.6081	0.5191	0.1828	0.7701		0.8862	0.7784	**0.0008**
				shoot	0.6690	0.5981	0.4048	0.7432	0.8862		0.7423	**0.0301**
				legume	0.9941	0.9113	0.6986	0.9584	0.7784	0.7423		0.0781
				seed	**0.0018**	**0.0095**	**0.0070**	**0.0194**	**0.0008**	**0.0301**	0.0781	

F – F-ratio

p – probability

values <0.05 are shown in **bold**

Table S6. Results of one-way ANOVA of differences between BC_{F1} values of PHEs in investigated regions of southern Poland.

PHE	F	p	Confidence interval	Fisher's LSD test, probabilities for post-hoc tests				
**As**	2.3622	0.0739	0.95	Non-significant differences				
**Cd**	4.9854	**0.0026**	0.95	Error: between MS=0.01170, df=140.000				
					{1}	{2}	{3}	{4}
					0.03273	0.04120	0.11892	0.08577
				Opolskie		0.7402	**0.0009**	**0.0393**
				Śląskie	0.7402		**0.0027**	0.0826
				Małopolskie	**0.0009**	**0.0027**		0.1957
				Świętokrzyskie	**0.0393**	0.0826	0.1957	
**Co**	8.8440	**0.000021**	0.95	Error: between MS=0.00018, df=140.000				
					{1}	{2}	{3}	{4}
					0.00277	0.00237	0.01508	0.00078
				Opolskie		0.89884	**0.00014**	0.52796
				Śląskie	0.89884		**0.00009**	0.61412
				Małopolskie	**0.00014**	**0.00009**		**0.00001**
				Świętokrzyskie	0.52796	0.61412	**0.00001**	
**Cu**	1.0174	0.3870	0.95	Non-significant differences				
**Ni**	0.8124	0.4890	0.95	Non-significant differences				
**Pb**	3.0255	**0.0317**	0.95	Error: between MS=1.1451, df=140.000				
					{1}	{2}	{3}	{4}
					0.42949	0.05467	0.81446	0.44124
				Opolskie		0.1395	0.1292	0.9629
				Śląskie	0.1395		**0.0031**	0.1276
				Małopolskie	0.1292	**0.0031**		0.1412
				Świętokrzyskie	0.9629	0.1276	0.1412	
**Sb**	6.4694	**0.000393**	0.95	Error: between MS=0.01044, df=140.00				
					{1}	{2}	{3}	{4}
					0.09085	0.02089	0.07645	0.00058
				Opolskie		**0.0043**	0.5508	**0.0003**
				Śląskie	**0.0043**		**0.0225**	0.4006
				Małopolskie	0.5508	**0.0225**		**0.0020**
				Świętokrzyskie	**0.0003**	0.4006	**0.0020**	
**Tl**	0.6551	0.5810	0.95	Non-significant differences				
**Zn**	4.4558	**0.0051**	0.95	Error: between MS=0.12518, df=140.00				
					{1}	{2}	{3}	{4}
					0.07802	0.02284	0.20467	0.29881
				Opolskie		0.5092	0.1311	**0.0090**
				Śląskie	0.5092		**0.0309**	**0.0012**
				Małopolskie	0.1311	**0.0309**		0.2609
				Świętokrzyskie	**0.0090**	**0.0012**	0.2609	

F – F-ratio

p – probability

values <0.05 are shown in **bold**

Table S7. Results of one-way ANOVA of differences between average BC_{EDTA} values of PHEs in groups of vegetables.

PHE	F	p	Confidence interval	Fisher's LSD test, probabilities for post-hoc tests								
**Cd**	2.4986	**0.0191**	0.95	Error: between MS=0.03309, df=136.000								
					{1}	{2}	{3}	{4}	{5}	{6}	{7}	{8}
					0.14508	0.08828	0.18766	0.04218	0.02745	0.00139	0.05725	0.07301
				root		0.3209	0.3267	0.1605	**0.0289**	0.1418	0.3680	0.2083
				tuber	0.320892		0.0606	0.5594	0.3205	0.3943	0.7607	0.8127
				leaf	0.326681	0.0606		**0.0381**	**0.0012**	0.0511	0.1706	**0.0307**
				inflorescence	0.160541	0.5594	**0.0381**		0.8468	0.7148	0.8926	0.6961
				fruit	**0.028871**	0.3205	**0.0012**	0.8468		0.7940	0.7653	0.4565
				shoot	0.141778	0.3943	0.0511	0.7148	0.7940		0.6648	0.4824
				legume	0.367969	0.7607	0.1706	0.8926	0.7653	0.6648		0.8770
				seed	0.208350	0.8127	**0.0307**	0.6961	0.4565	0.4824	0.8770	
**Cu**	1.8250	0.0872	0.95	Non-significant differences								
**Ni**	1.3224	0.2443	0.95	Non-significant differences								
**Pb**	2.1193	**0.0455**	0.95	Error: between MS=0.00003, df = 136.000								
					{1}	{2}	{3}	{4}	{5}	{6}	{7}	{8}
					0.00126	0.00148	0.00296	0.0000	0.00252	0.00058	0.01011	0.00092
				root		0.8893	0.1637	0.5411	0.4012	0.8047	**0.0015**	0.8362
				tuber	0.8893		0.3181	0.5051	0.5464	0.7532	**0.0030**	0.7592
				leaf	0.1637	0.3181		0.1320	0.7458	0.3728	**0.0081**	0.1703
				inflorescence	0.5411	0.5051	0.1320		0.2414	0.8535	**0.0016**	0.6770
				fruit	0.4012	0.5464	0.7458	0.2414		0.4902	**0.0076**	0.3548
				shoot	0.8047	0.7532	0.3728	0.8535	0.4902		**0.0094**	0.9041
				legume	**0.0015**	**0.0030**	**0.0081**	**0.0016**	**0.0076**	**0.0094**		**0.0016**
				seed	0.8362	0.7592	0.1703	0.6770	0.3548	0.9041	**0.0016**	
**Zn**	1.7471	0.1031	0.95	Non-significant differences								

F – F-ratio

p – probability

values <0.05 are shown in **bold**

Table S8. Results of one-way ANOVA of differences between BC_{EDTA} values of PHEs in investigated regions of southern Poland.

PHE	F	p	Confidence interval	Fisher's LSD test, probabilities for post-hoc tests				
**Cd**	5.9461	**0.00076**	0.95	Error: between MS=0.03218, df=140.000				
					{1}	{2}	{3}	{4}
					0.00000	0.00093	0.00170	0.00114
				Opolskie		0.3337	**0.0005**	**0.0019**
				Śląskie	0.3337		**0.0099**	**0.0296**
				Małopolskie	**0.0005**	**0.0099**		0.6774
				Świętokrzyskie	**0.0019**	**0.0296**	0.6774	
**Cu**	2.4120	0.0694	0.95	Non-significant differences				
**Ni**	0.8308	0.4790	0.95	Non-significant differences				
**Pb**	5.3714	**0.0016**	0.95	Error: between MS=0.00003, df=140.000				
					{1}	{2}	{3}	{4}
					0.00185	0.00035	0.00489	0.00148
				Opolskie		0.2065	**0.0113**	0.7558
				Śląskie	0.2065		**0.0002**	0.3400
				Małopolskie	**0.0113**	**0.0002**		**0.0046**
				Świętokrzyskie	0.7558	0.3400	**0.0046**	
**Zn**	5.1167	**0.0022**	0.95	Error: between MS=1.0179, df=140.00				
					{1}	{2}	{3}	{4}
					0.1409	0.0670	0.4794	0.8997
				Opolskie		0.7566	0.1567	**0.0017**
				Śląskie	0.7566		0.0851	**0.0006**
				Małopolskie	0.1567	0.0851		0.0794
				Świętokrzyskie	**0.0017**	**0.0006**	0.0794	

F – F-ratio

p – probability

values <0.05 are shown in **bold**

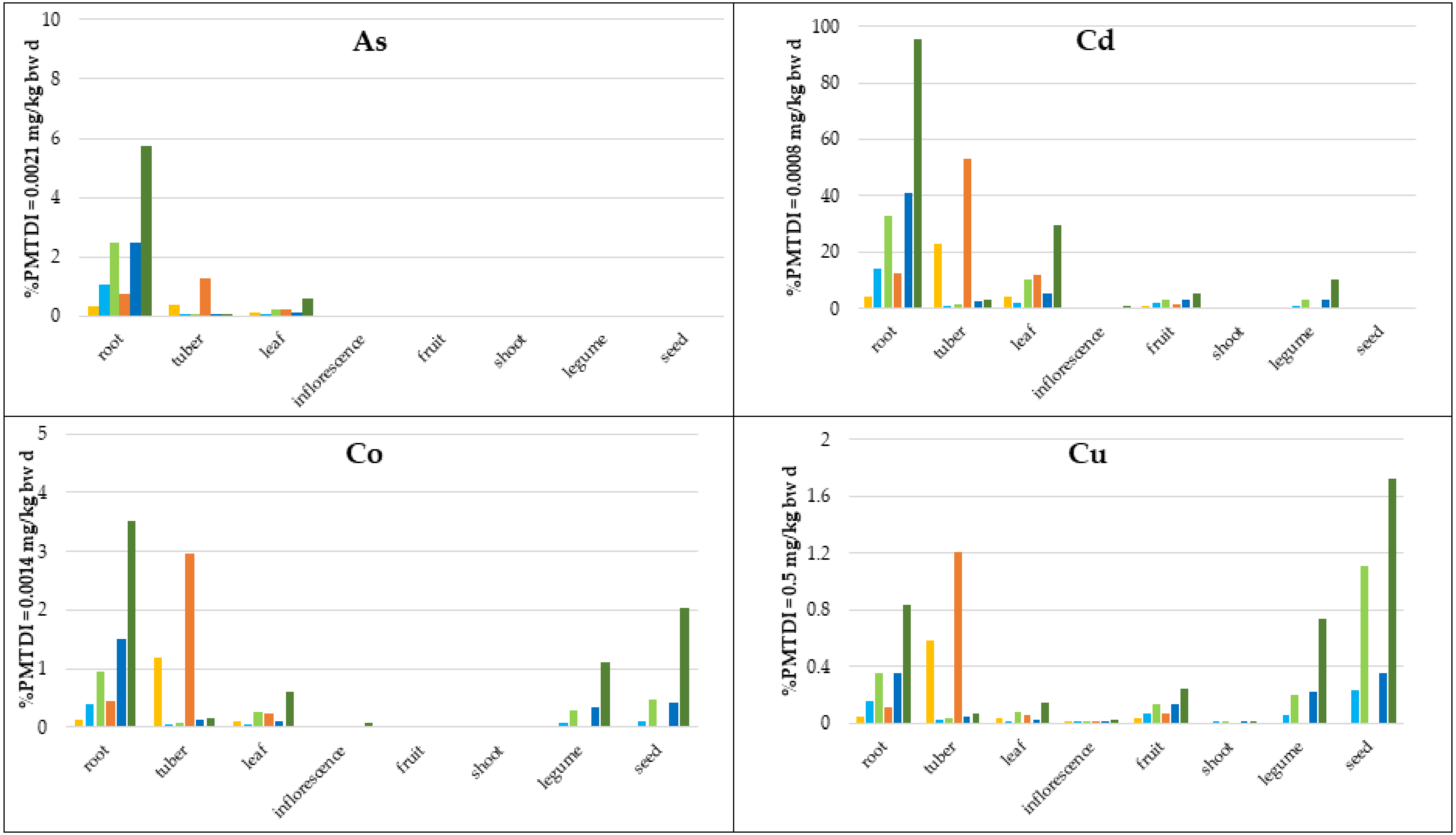
As
%PMTDI = 0.0021 mg/kg bw d
Cd
%PMTDI = 0.0008 mg/kg bw d
Co
%PMTDI = 0.0014 mg/kg bw d
Cu
%PMTDI = 0.5 mg/kg bw d
root
tuber
leaf
inflorescence
fruit
shoot
legume
seed

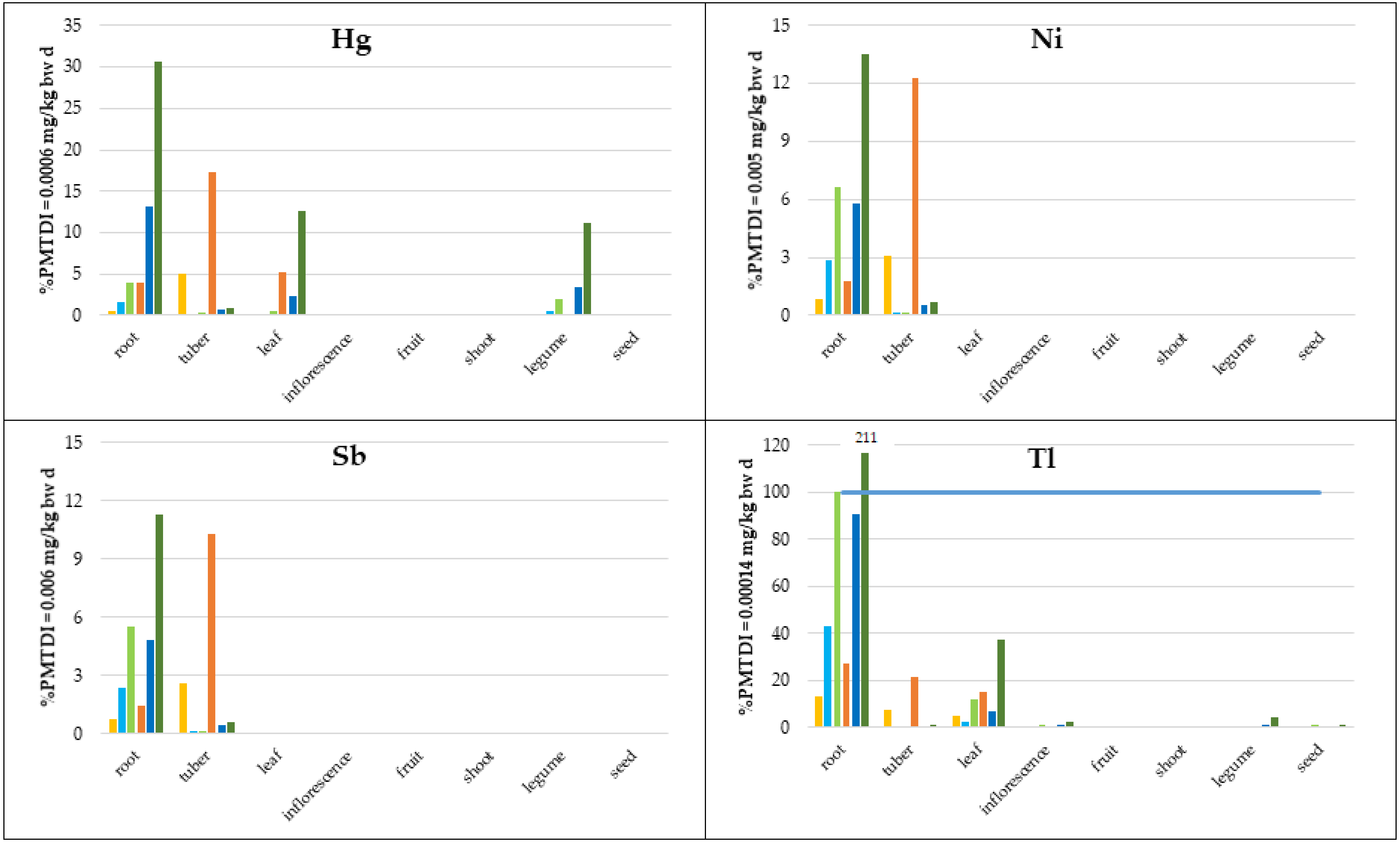
Hg
%PMTDI =0.0006 mg/kg bw d
35
30
25
20
15
10
5
0
root
tuber
leaf
inflorescence
fruit
shoot
legume
seed
Ni
%PMTDI =0.005 mg/kg bw d
15
12
9
6
3
0
Sb
%PMTDI =0.006 mg/kg bw d
Tl
%PMTDI =0.00014 mg/kg bw d
211
120
100
80
60
40
20
0

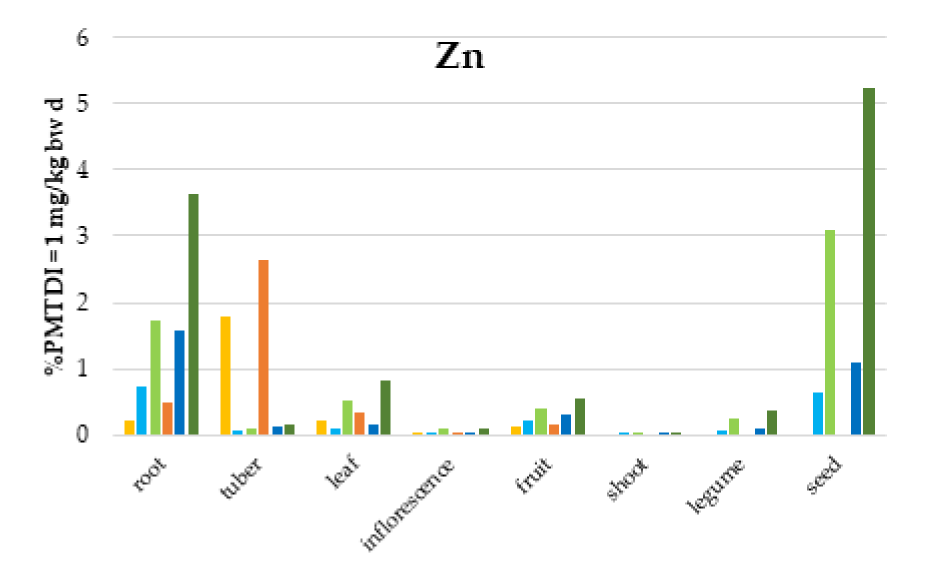

mean adult PL
mean adult USEPA
mean child USEPA
P95 adult PL
P95 adult USEPA
P95 child USEPA
100%PMTDI

mean adult PL – intake rate for adults, statistical data for Poland, mean PHEs concentration

mean adult USEPA – intake rate for adults, USEPA recommended values, mean PHEs concentration

mean child USEPA – intake rate for children, USEPA recommended values, mean PHEs concentration

P95 adult PL – intake rate for adults statistical data for Poland 95th percentile of PHEs concentration

P95 adult USEPA – intake rate for adults, USEPA recommended values, 95th percentile of PHEs concentration

P95 child USEPA – intake rate for children, USEPA recommended values, 95th percentile of PHEs concentration

Figure S3. Daily intake rates of PHEs via consumed vegetables, as a percentage of provisional maximum tolerable daily intake (%PMTDI)

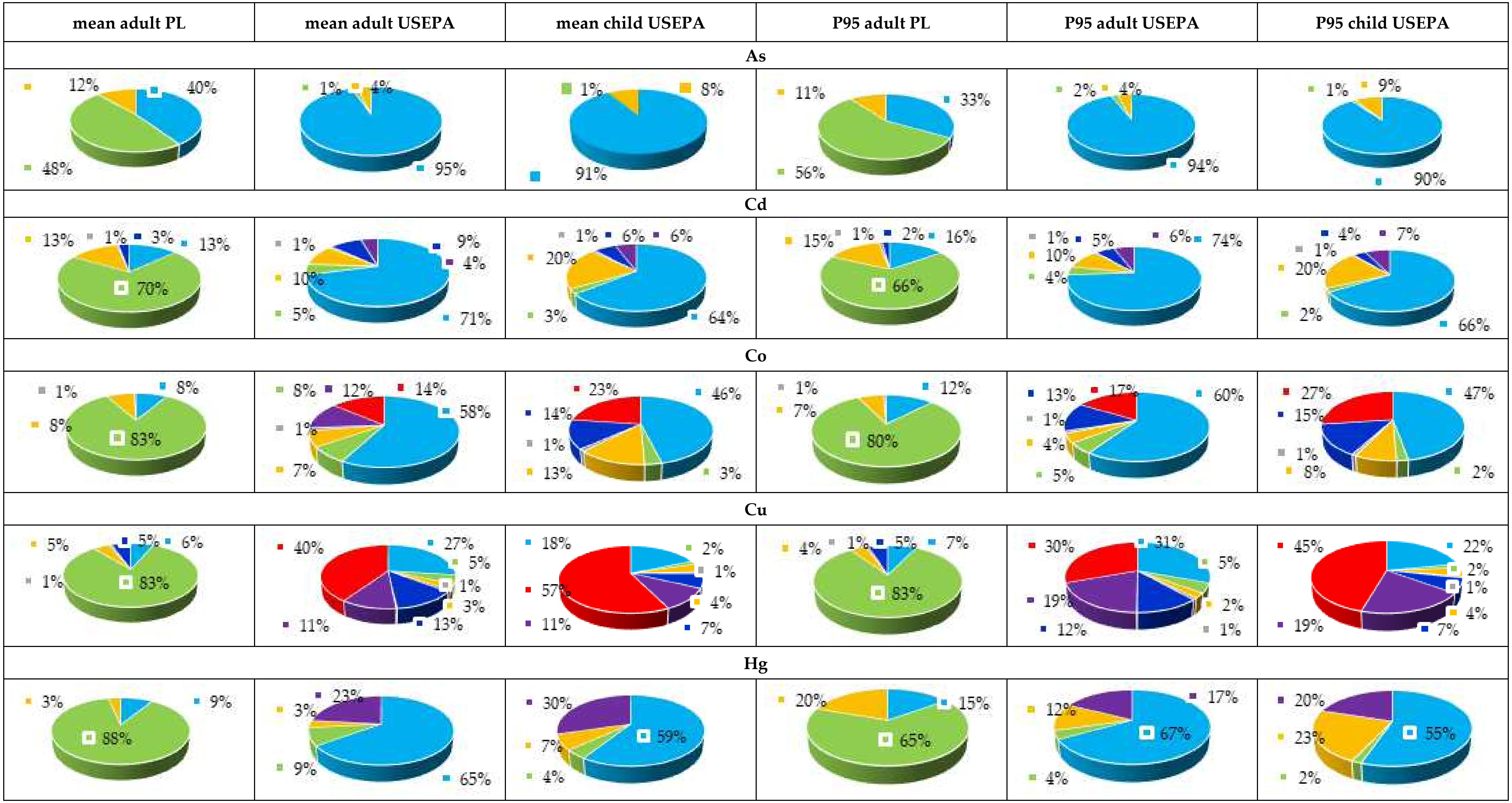
mean adult PL
mean adult USEPA
mean child USEPA
P95 adult PL
P95 adult USEPA
P95 child USEPA
As
12% 40% 48%
1% 4% 95%
1% 8% 91%
11% 33% 56%
2% 4% 94%
1% 9% 90%
Cd
13% 1% 3% 13% 70%
1% 9% 4% 10% 5% 71%
1% 6% 6% 20% 3% 64%
15% 1% 2% 16% 66%
1% 5% 6% 74% 10% 4%
4% 7% 1% 20% 2% 66%
Co
1% 8% 8% 83%
8% 12% 14% 58% 1% 7%
23% 46% 14% 1% 13% 3%
1% 12% 7% 80%
13% 17% 60% 1% 4% 5%
27% 47% 15% 1% 8% 2%
Cu
5% 5% 6% 1% 83%
40% 27% 5% 1% 3% 11% 13%
18% 2% 1% 57% 4% 11% 7%
4% 1% 5% 7% 83%
30% 31% 5% 19% 2% 12% 1%
45% 22% 2% 1% 4% 19% 7%
Hg
3% 9% 88%
23% 3% 9% 65%
30% 7% 59% 4%
20% 15% 65%
17% 12% 67% 4%
20% 23% 55% 2%

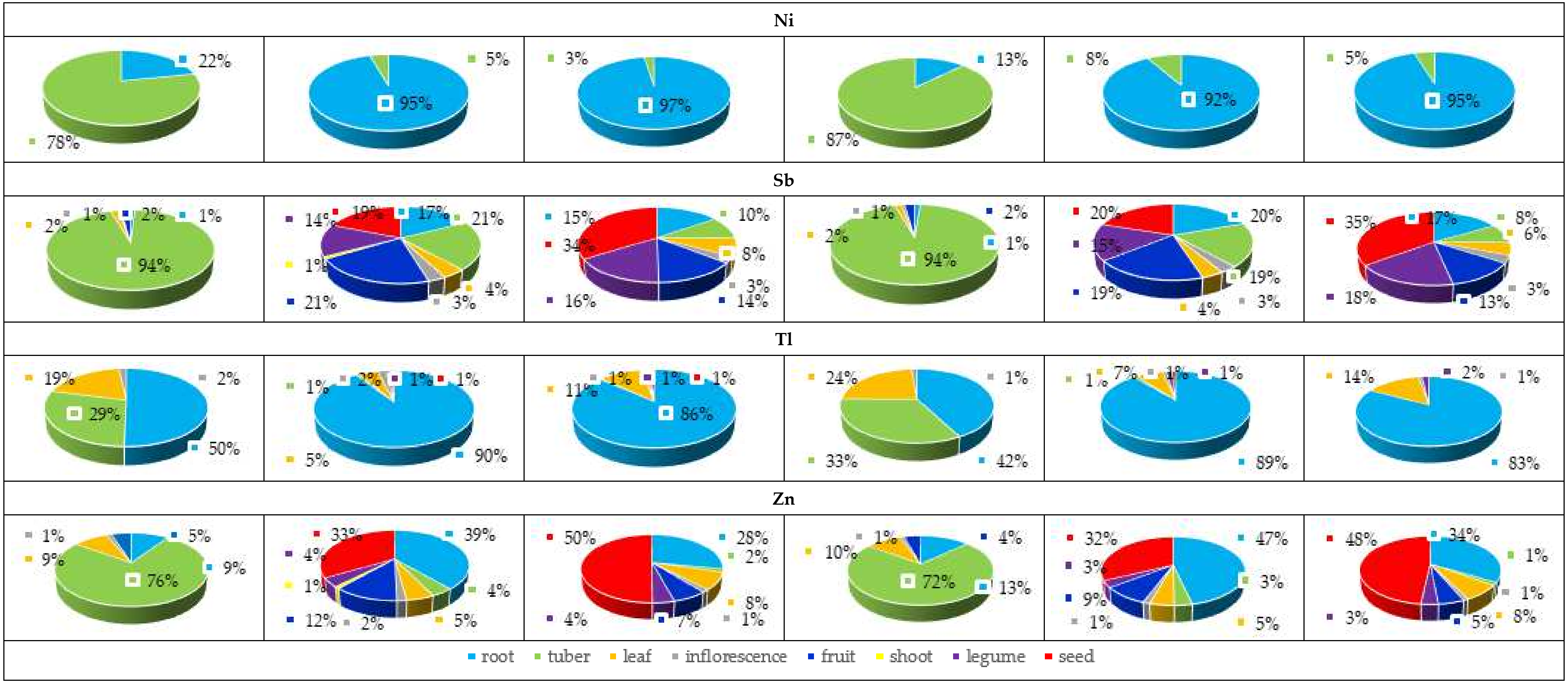

mean adult PL – intake rate for adults, statistical data for Poland, mean PHEs concentration; mean adult USEPA – intake rate for adults, USEPA recommended values, mean PHEs concentration, mean child USEPA – intake rate for children, USEPA recommended values, mean PHEs concentration; P95 adult PL – intake rate for adults statistical data for Poland 95th percentile of PHEs concentration; P95 adult USEPA – intake rate for adults, USEPA recommended values, 95th percentile of PHEs concentration
P95 child USEPA – intake rate for children, USEPA recommended values, 95th percentile of PHEs concentration

Figure S4. The contribution of various groups of vegetables to the PHE daily intake rates

© 2019 by the authors. Submitted for possible open access publication under the terms and conditions of the Creative Commons Attribution (CC BY) license (http://creativecommons.org/licenses/by/4.0/).
